# Leveraging Ensemble Machine Learning Models for the Detection of Primary Myelofibrosis in Electronic Health Records

**DOI:** 10.3390/cancers18101618

**Published:** 2026-05-16

**Authors:** Arkadiusz Sycz, Michal J. Dabrowski, Kinga Marciniak, Aleksandra Jurczuk, Anna Meryn, Michał Konopelko, Marek Dudziński, Mirosław Markiewicz, Wojciech Homenda, Marta Sobas, Łukasz Szukalski, Karolina Kaczorowska-Bilska, Agnieszka Gala-Błądzińska, Szymon Fornagiel, Jarosław Piszcz, Grzegorz Helbig, Patryk Węglarz, Sylwia Kot, Paweł Turczyn, Brygida Kwiatkowska, Marcin Rymko, Artur Przelaskowski, Grzegorz W. Basak, Karol Lis

**Affiliations:** 1 Saventic Health, Polna 66/12 Street, 87-100 Torun, Poland; m.dabrowski@ipipan.waw.pl (M.J.D.); kinga.marciniak@saventic.com (K.M.); aleksandra.jurczuk@saventic.com (A.J.); anna.meryn@saventic.com (A.M.); michal.konopelko@saventic.com (M.K.); marek.dudzinski@saventic.com (M.D.); grzegorz.basak@wum.edu.pl (G.W.B.); 2 Faculty of Mathematics and Information Science, Warsaw University of Technology, St. Koszykowa 75, 00-662 Warsaw, Poland; artur.przelaskowski@pw.edu.pl; 3 Computational Biology Group, Institute of Computer Science of the Polish Academy of Sciences, 01-248 Warsaw, Poland; 4 The HCP Hospital in Poznan, St. 28 Czerwca 1956 r. 194, 61-485 Poznan, Poland; 5 Department of Clinical Immunology, Medical University of Bialystok, 15-274 Bialystok, Poland; 6 Skåne University Hospital, 214 28 Malmö, Sweden; 7 Department of Hematology, F. Chopin University Clinical Hospital in Rzeszów, 35-055 Rzeszow, Poland; mmarkiewicz@ur.edu.pl (M.M.); ksylwia13@gmail.com (S.K.); 8 Department of Hematology, Faculty of Medicine, Collegium Medicum, University of Rzeszów, 35-959 Rzeszow, Poland; 9 Regional Hospital of Slupsk and Institute of Health Sciences, Pomeranian University of Slupsk, 76-200 Slupsk, Poland; wojhom@sl.home.pl; 10 Department of Hematology, Jan Biziel University Hospital No. 2, 85-163 Bydgoszcz, Poland; marta.sobas@cm.umk.pl (M.S.); karolina.kaczorowska@cm.umk.pl (K.K.-B.); 11 Department of Hematology, Collegium Medicum in Bydgoszcz, Nicolaus Copernicus University in Toruń, 85-067 Bydgoszcz, Poland; lukasz.szukalski@cm.umk.pl; 12 Department of Nephrology and Dialysis Unit, University Clinical Hospital, Fryderyka Chopina 2, 35-055 Rzeszow, Poland; aggala@ur.edu.pl; 13 Department of Nephrology and Endocrinology, Faculty of Medicine, University of Rzeszów, 35-959 Rzeszow, Poland; 14 Jędrzej Śniadecki Specialist Hospital, 33-300 Nowy Sacz, Poland; szf@wp.pl; 15 Department of Haematology, Internal Medicine and Angiology with Haematopoietic Cell Transplantation Unit, Medical University of Białystok, 15-276 Bialystok, Poland; jaroslawpiszcz@gmail.com; 16 Department of Hematology and Bone Marrow Transplantation, Medical University of Silesia, 40-514 Katowice, Poland; ghelbig@sum.edu.pl (G.H.); patryk.weglarz@sum.edu.pl (P.W.); 17 National Institute of Geriatrics, Rheumatology and Rehabilitation, Clinic of Early Arthritis, 02-637 Warsaw, Poland; pawel.turczyn@spartanska.pl (P.T.); brygida.kwiatkowska@spartanska.pl (B.K.); 18 Department of Hematology, L. Rydygier Regional Hospital in Torun, 87-100 Torun, Poland; marcin.rymko@wszz.torun.pl; 19 Department of Hematology, Transplantation and Internal Medicine, Medical University of Warsaw, 02-097 Warsaw, Poland

**Keywords:** primary myelofibrosis, rare disease, classification, class imbalance, AutoML, gradient boosting trees, positive-unlabeled

## Abstract

**Background and Objectives**: Primary myelofibrosis (PMF) is a rare hematologic malignancy with non-specific symptoms, causing diagnostic delays and missed diagnoses. Automated screening in heterogeneous electronic health records (EHRs) is challenging due to class imbalance, data sparsity, and incomplete labeling. We investigated two complementary objectives: (1) developing a screening algorithm using routine EHR data to identify PMF-risk patients for hematology consultation, and (2) assessing the applicability of positive-unlabeled (PU) learning. **Methods**: Using EHR data from 10 Polish hospitals, we evaluated several ensemble models and found that LightGBM achieved the best performance. **Results**: LightGBM achieved AP 20.83% (95% CI: 19.18–24.35%), sensitivity 45.52% (95% CI: 39.48–52.72%), and precision 14.72% (95% CI: 13.80–17.01%)—substantially exceeding expected prevalence (over 340-fold enrichment). The model captured interactions among RDW and PLT parameters revealing high-risk unlabeled patients with confirmed diagnoses (*n* = 5) or clinical profiles resembling PMF cases (*n* = 31), confirming PU problem. **Conclusions:** Although PU methods enhanced sensitivity, they reduced precision to levels exceeding real-world healthcare capacity. Ensemble learning enables disease identification in clinical settings.

## Simple Summary

Primary myelofibrosis is a rare blood cancer that is often diagnosed late because early symptoms can mimic other conditions. We explored whether statistical models can scan routine hospital records to find patients who need a specialist assessment. Using data from ten hospitals in Poland, we compared several model types and tested methods designed for situations where some patients have no confirmed diagnosis label. The best approach found meaningful patterns in common blood test results and identified 46% of known confirmed patients and 5 confirmed out of 150 unlabeled patients. These results suggest that practical, low-cost data tools can support detection of rare diseases and help researchers design better screening strategies in real clinical settings.

## 1. Introduction

The application of machine learning (ML) in healthcare, particularly in leveraging electronic health records (EHR) for disease prediction, represents a rapidly expanding research frontier [1,2,3]. This is especially pertinent for rare diseases, where early and accurate diagnosis remains a significant challenge due to non-specific symptoms, low prevalence, and fragmented patient data across the healthcare system. Within this domain, various algorithms have been evaluated, including logistic regression, support vector machines, random forests, neural networks, and deep learning techniques. Among these, XGBoost, an ensemble method, consistently demonstrates superior predictive performance and is widely adopted in diagnostic prediction studies [4,5,6,7,8]. Its efficacy stems from its robust handling of large, complex, and often incomplete datasets, coupled with effective regularization strategies. While advanced methods like large language models (LLMs) are emerging for tasks such as information extraction and clinical decision support, their real-world implementation in healthcare remains challenging. Key barriers include high computational demands and data privacy regulations, such as the General Data Protection Regulation (GDPR) in Europe and the Health Insurance Portability and Accountability Act (HIPAA) in the United States. These regulations often preclude the use of external cloud services. Consequently, LLM deployment typically requires resource-intensive on-premises infrastructure that many healthcare institutions lack. This creates a gap between the transformative potential of LLMs and their practical, scalable use. Therefore, efficient and less computationally demanding ML approaches remain indispensable for initial patient screening and risk stratification. These methods help identify at-risk patients for subsequent evaluation with more resource-intensive LLM-based systems. Studies highlight the promise of ML in hematology. For instance, Kimura K. et al. [5] developed an XGBoost model that achieved over 90% sensitivity and specificity in distinguishing between polycythemia vera (PV), essential thrombocythemia (ET), and primary myelofibrosis (PMF) using comprehensive morphological and complete blood count (CBC) features. In another study, Radhakrishnan et al. [6] reported a robust predictive model for myelodysplastic syndromes (MDS), achieving 79% sensitivity, 80% specificity, and an AUROC of 87%, utilizing demographic data, lab results, vital signs, and diagnostic codes from EHRs. Similarly, Hauser et al. [8] demonstrated the high predictive power of ML for chronic myeloid leukemia (CML) based on CBC data, achieving AUROCs ranging from 87% to 96% even in a highly imbalanced dataset, where only 6.1% of cases were positive diagnoses. These examples illustrate the significant diagnostic potential of ML models, particularly XGBoost, in complex hematological conditions. However, conventional supervised learning often faces limitations in performance when applied to real-world EHR data, particularly due to high data sparsity and clinical heterogeneity. EHR data are heterogeneous, stemming from diverse sources, measurement tools, and time periods. They are frequently incomplete due to practical collection constraints [9,10,11,12,13]. They are also non-stationary, as medical guidelines evolve [14]. Moreover, they often reflect clinical decisions influenced by non-transparent physician reasoning [15,16] or personal experience rather than objective medical ontologies [17]. Unlike randomized controlled trials (RCTs), EHR data are observational and involve uncontrolled factors like selection bias [1,11,12]. Rare diseases, in particular, are often underrepresented. Patients with mild or atypical symptoms may delay or avoid diagnosis entirely, leading to a disproportionate concentration of diagnoses among those with more typical or severe manifestations.

Compounding this challenge, EHR datasets frequently contain only partially labeled data; while a subset of patients is definitively diagnosed with the target disease, the vast majority remain untested and lack definitive labels. Critically, EHR systems primarily record positive disease confirmations, but rarely include explicit diagnostic codes for ruled-out conditions. When a diagnosis is considered but not confirmed, this outcome is often not formally registered as a negation in the health record [18]. This absence of confirmed negative instances creates a significant obstacle for supervised learning, as constructing a reliable negative cohort is typically impractical, requiring exhaustive, time-consuming, and error-prone clinical review [19]. Without verified negative labels, traditional threshold-based evaluation metrics (e.g., sensitivity, specificity, precision) become difficult to interpret and may misrepresent true model performance, particularly in real-world deployment scenarios where disease prevalence is uncertain or underreported [20].

Positive-unlabeled (PU) learning is an ML paradigm closely related to binary classification, where the goal is to distinguish between positive and negative instances based on their attributes. Under PU learning, a subset of positive examples is labeled, while negative instances and the remaining positive ones are unlabeled. This allows the model to use unlabeled data to infer the underlying class distribution despite incomplete labeling. PU has been successfully applied in medicine, including brain tumor image segmentation [21], disease gene identification [22], and protein function prediction [23]. In these examples, unlabeled sets often included hidden positives, making PU frameworks well suited to scenarios where treating all unlabeled data as negative would introduce systematic error. We hypothesized that PU methodology would better adapt to real-world clinical modeling scenarios such as ours. We therefore adapted two foundational PU methods to enhance our ensemble models: the Elkan & Noto approach [24], used to estimate the true proportion of positives in the unlabeled set and calibrate classification outputs, and the Spy method [25], which embeds a subset of positive ‘spy’ samples into unlabeled data to identify reliable negatives. To our knowledge, this method has not yet been evaluated for rare-disease classification based on EHR data; testing it addresses a critical research gap.

This study evaluates the performance of tree-based ensemble models within two distinct classifier settings: (i) traditional binary classification and (ii) positive-unlabeled (PU) learning, incorporating established methods (Elkan and Noto, Spy). The work is driven by two complementary objectives. First, from a clinical perspective, we investigate whether it is feasible to develop an algorithm capable of screening patients using routinely collected electronic health records (EHR) data, in order to identify individuals at risk of primary myelofibrosis (PMF) who may benefit from further hematology consultation. Second, from a methodological perspective, we aim to assess the applicability and effectiveness of advanced machine learning strategies (particularly PU learning) in the context of rare disease detection in real-world EHR data.

We leverage EHR data from ten Polish hospitals, focusing on complete blood count (CBC) parameters and ICD-10 codes, to develop a predictive model for PMF. PMF is a rare hematologic disease representing a heterogeneous group of conditions in terms of underlying pathophysiology, symptom burden, and clinical presentation; its often nonspecific and variable manifestations make early recognition challenging, yet crucial for timely intervention and improved patient outcomes. Retrospective evaluation yielded an Average Precision (AP) of 20.83% (95% CI: 19.18–24.35%) for the best-performing model (LightGBM). Our findings demonstrate the potential of ensemble learning approaches, particularly in identifying rare diseases within complex, real-world clinical data environments.

## 2. Materials and Methods

### 2.1. Studied Population

**Figure 1:**
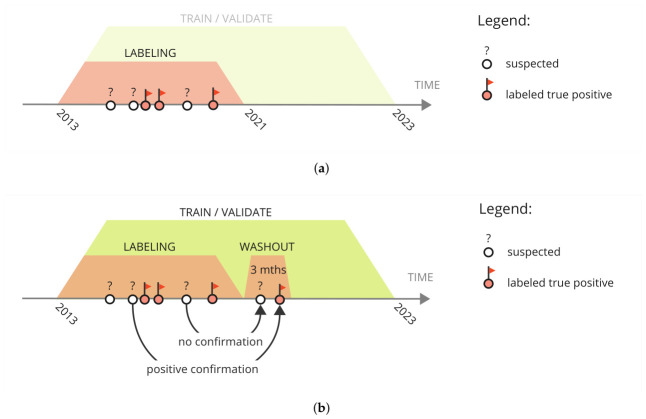
Identification and labeling of patients with suspected PMF. Circles on the time axis represent individual patients and their labeling status at a given time point. (**a**) Manual verification of EHRs for suspected patients within the labeling window (2013–2021, red shaded area); (**b**) Re-evaluation after a 3-month washout period for patients with updated records. The green shaded area indicates the time window (2013–2023) of data used for training and validation.

This study utilized fully anonymised EHRs collected from 10 medical centers in Poland, covering the period from 2013 to 2022 for retrospective analysis. The 10 participating hospitals represent a diverse cross-section of the Polish healthcare system. They include both specialized hematology centers and general regional hospitals, which introduces significant challenges in data normalization and record completeness. Data were sourced from the Saventic Health database, encompassing a comprehensive array of medical information: laboratory test results, diagnosis, ICD-10 codes, year of birth, gender, reported symptoms, descriptive texts of medical history, visit records, imaging studies descriptions, and epicrisis. We identified putative PMF patients through a two-step process. First, patients were selected if their EHR contained the phrase “myelofibrosis” or its synonyms (identified using the regex: \b[mM]ielofibroz) in any text description, or if they had one of the specific ICD-10 codes: C94, C94.4, D47, D47.1, and D47.4. Second, the selected EHRs underwent manual review by experienced physicians. This review categorized patients into three groups: (i) “confirmed PMF” (PMFc) for those with a definitive diagnosis; (ii) “suspected PMF” if a final diagnosis of PMF was absent but the EHR indicated unexplained symptoms consistent with PMF [26,27]; and (iii) “excluded” if the diagnostic process led to an alternative diagnosis (Figure 1a). This initial labeling was completed in August 2021. To ensure diagnostic stability, all patients initially labeled as “suspected” underwent a re-evaluation after a 3-month washout period. If their PMF diagnosis was subsequently confirmed, they were then included in the PMFc group (Figure 1b). The control group (Cr) consisted of patients from the same 2013–2022 period, who did not have any ICD-10 codes or regex matches related to the PMF positive group. From over 3 million patients, we selected a total of 110,000 Cr patients, with 11,000 chosen from each of 10 medical centers. Recognizing that the PMFc cohort predominantly consisted of advanced-age individuals, we performed stratified sampling to age-match and gender-match the control group to the PMFc group. This approach was crucial to minimize potential confounding effects and enhance the internal validity of our results. However, it is important to acknowledge that this matching strategy resulted in a control group composition that deviates from the general population distribution, potentially limiting the direct generalizability of our findings to the broader population.

### 2.2. Data Extraction, Preprocessing, and Statistical Analysis

All data extraction and subsequent analyses were conducted using Python 3.8 and a suite of key libraries, including kedro [28] (data pipelines), SQLAlchemy [29] (database access and object-relational mapping), pandas [30] (tabular data processing), numpy [31] (numerical computing), statsmodels [32] (statistical modeling), and scikit-learn [33] (machine learning utilities). We extracted the following information for both PMF confirmed cases (PMFc) and control group (Cr) patients: year of birth, gender, details about visits, at least one ICD-10 code, and 22 CBC parameters. To enrich the dataset, extracted ICD-10 codes were mapped to higher-level phenotypes using the PheWAS (phenome-wide association studies) system [34]. To rigorously prevent label leakage, specific ICD-10 codes highly indicative of hematologic malignancies (D45, D46, D47, D70-D77, C81-C96) were excluded from the feature set. Moreover, histopathological features from bone marrow biopsy were intentionally excluded. Since bone marrow biopsy is a specialized procedure typically performed after hematological referral, incorporating such data would limit the model’s utility to a later diagnostic stage. For PMFc patients, medical records from the two years preceding their diagnosis were analyzed. For Cr patients, a corresponding two-year time window was randomly selected from their EHR data, ensuring it ended no later than their last visit. For some Cr patients, this randomly selected window might extend before their first recorded visit, potentially resulting in a time window shorter than two years. For patients with multiple measurements of a single parameter, values were aggregated. Floating-point parameters were summarized into eight features: the 25th, 50th, and 75th percentiles, minimum, maximum, variance, and absolute difference between the first and last parameter measurements, and monotonicity of change (“True” = increase, “False” = decrease). Binary variables represented the presence or absence of specific conditions within the two-year time window. Next, continues features were discretized into 10 intervals using percentile-based binning. To reflect typical physiological ranges, bin boundaries were determined based on the vast and diverse control group (Cr). Phenotypes, ICD-10 codes, and non-mandatory CBC results were included in the final feature set only if they were present independently in at least 5% of both the Cr and PMFc patient cohorts. No other preprocessing steps were performed. All built-in model feature selection and tuning were performed strictly within the training folds of the cross-validation process. Each record was associated with the patient’s age at the time the corresponding measurement was taken.

For univariate analysis, a logistic regression model with balanced class weighting was utilized, implemented via the statsmodels package [32]. This approach was specifically chosen to account for the substantial class imbalance between the PMFc cases and the Cr. For each evaluated feature, we calculated *p*-values, alongside Odds Ratios (OR) to quantify the effect size. To rigorously control for the false discovery rate (FDR) across multiple comparisons, the Benjamini–Hochberg procedure was applied. An FDR-adjusted *p*-value of less than 0.05 was considered statistically significant. Finally, a volcano plot was generated to simultaneously visualize the statistical significance and the magnitude of the effect (OR) for each evaluated parameter.

### 2.3. Model Training and Evaluation

We compared two classification paradigms: (i) traditional supervised classification (PMFc as positive, Cr as negative) and (ii) semi-supervised positive-unlabeled (PU) learning (PMFc labeled positive, Cr as unlabeled mixture of both positive and negative samples). We evaluated four gradient-boosted ensemble algorithms: XGBoost [35], LightGBM [36], and CatBoost [37], alongside Random Forest [38] (with Weight of Evidence transformation [39]). These models are widely recognized as state-of-the-art for tabular data modeling, often outperforming deep learning architectures due to their superior handling of non-linear relationships, data sparsity, and various feature scales without requiring extensive normalization [40,41]. Hyperparameter configuration was established via Bayesian optimization [42], maximizing Average Precision (AP) for the PMFc class across 50 iterations using the available cohort within a 10-fold cross-validation framework (10 repetitions) to identify robust global parameters. To prevent overfitting given the extremely small positive cohort, we applied strong regularization and strictly limited both the maximum depth and the total number of trees (Supplementary Table S1 contains the parameter constraints). No synthetic data generation or imputation was used to preserve fidelity with real-world clinical conditions and ensure interpretability [10,43,44]. For PU learning, we implemented the Elkan & Noto method [24] and the Spy method [45] (Supplement—Details of PU methods). The Elkan & Noto method used a 10% hold-out set to calibrate classification probabilities. In the Spy method, 10% of positive samples were added as “spies” to the unlabeled set to identify reliable negatives based on their scores, assuming a 20% noise tolerance threshold.

The resulting hyperparameter configurations were evaluated using repeated randomized stratified 10-fold cross-validation (10 repetitions), yielding a total of 100 independent validation runs. Metrics included Average Precision (AP), Area Under the Receiver Operating Characteristic Curve (AUROC), F1 score, precision, recall, and specificity. Given the extreme class imbalance and the inherent lack of verified negative labels in the PU setting, we prioritized AP as a more reliable, threshold-agnostic measure of the precision–recall trade-off compared to standard interpolated PRC (precision–recall curve), as further detailed in the Discussion. For the top-performing model, we conducted an interpretability analysis. We also evaluated the number of patients who were classified correctly or incorrectly in more than half of the validation folds (referred to as frequently misclassified). These results are summarized in a Venn diagrams comparing all variants of the best-performing model.

## 3. Results

### 3.1. Patient Cohorts and Data Characterization

The patient selection process began with an initial cohort of 448 patients with suspected myelofibrosis based on ICD-10 codes and the term “myelofibrosis” in EHR text descriptions. Following a manual review by physicians, this group was narrowed down to 233 PMF confirmed cases (PMFc). Of these, 67 PMFc patients with a complete medical history and pre-diagnosis CBC results were selected for the final analysis (Table 1). The control group (Cr) consisted of 110,000 patients, selected through age- and gender-stratified sampling to match the demographic distribution of the PMFc cohort (Table 1).

**Figure 2:**
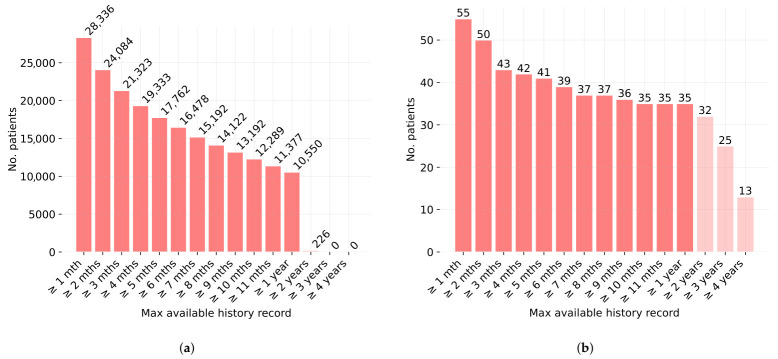
Number of patients with documented medical history of varying duration (months/years), collected either before diagnosis for PMFc (**a**) or before the end of the time window for Cr (**b**) (dark red– monthly, light red—yearly).

Analysis of EHR data completeness revealed a robust feature set for modeling; while record completeness was high (95.66% in PMFc and 88.91% in Cr), the key information for modeling was their temporal distribution. The EHRs of PMFc patients spanned over a year for more than half of the cohort and at least two years for nearly as many, providing a sufficiently long clinical history for analysis (Figure 2a). The pattern of increasingly frequent patient visits supports the use of longitudinal EHR data by reflecting a real-world disease trajectory rather than a random snapshot in time. In the one to two years preceding diagnosis, the average interval between visits exceeded 36 days. In the year preceding diagnosis, the average number of visits exceeded five, with intervals longer than 25 days. In the Cr group, a sharp decline was observed in the number of patients with data spanning more than one year (Figure 2b). Before the end of the time window, visit frequency averaged over three visits, spaced more than 21 days apart.

Ultimately, each patient in the final dataset was described by a comprehensive set of 212 features, including laboratory CBC parameters (*n* = 22) and their aggregations (*n* = 176), common phenotypes (*n* = 5), and ICD-10 codes (*n* = 7) (Supplementary Table S2). Following univariate analysis, we found that the demographic features of both cohorts did not differ significantly, likely due to our sampling strategy, whereas features related to the number of visits differed significantly but were not used for modeling to prevent potential label leakage (Table 2). As expected, based on median CBC values, patients with PMFc were significantly more anemic (lower HGB, HCT, and RBC values) and had higher PLT counts than the Cr group. Red cell indices, MCH and MCHC, were lower in PMFc, suggesting red blood cell hypochromia, while higher RDW indicated a less homogeneous red blood cell population. Platelet-related indices such as MPV, PDW, and PLCR were all significantly higher in the PMFc cohort. Interestingly, PMFc patients did not differ significantly in their white blood cell parameters (WBC, NEUT, LYMP, MONO), apart from absolute BASO level and lymphocyte percentage values (Table 2).

**Figure 3:**
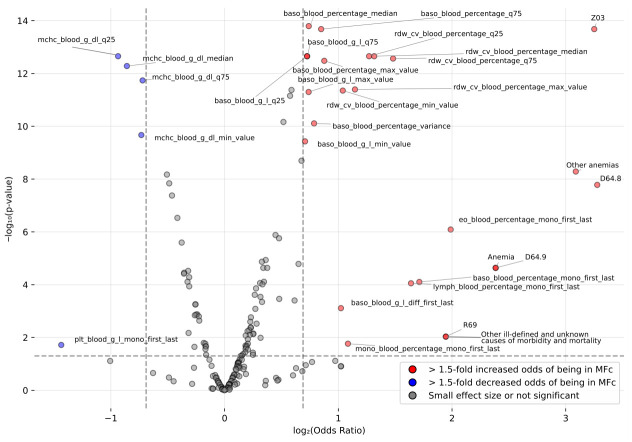
Volcano plot showing the modeled feature differences. The vertical lines indicate $\log_{2}\left(\mathrm{OR}\right)=\pm 0.585$, corresponding to an odds ratio of 1.5 and 0.67. The horizontal line represents a *p*-value threshold of 0.05, which corresponds to −log_10_ (*p*-value) = 1.3.

Next, a volcano plot was used to visualize and further assess the differences between cohorts by simultaneously evaluating statistical significance and the magnitude of effect (odds ratio, OR) (Figure 3). The analysis confirmed that while MCHC and its aggregates, as well as the direction of change in platelet count between the first and last measurement, had a lower OR in PMFc, the majority of significant and strong effect features had an increased OR (>1.5-fold increased odds). The highest increases were observed for phenotypes and ICD-10 codes, with “Other anemias” (D64.8) and “Observation for suspected disease” (Z03) showing over eight times higher odds in the PMFc cohort. Among CBC parameters, the strongest effect was observed for the change in eosinophils expressed as a percentage, with a fourfold odds increase. More moderate increases, closer to twofold, were observed for basophils, basophil aggregates, RDW, RDW aggregates, and monocytes (Figure 3).

### 3.2. Model Performance and Evaluation

To identify the most effective predictive strategy for detecting myelofibrosis patients, we compared the performance of traditional binary classification and various PU (positive-unlabeled) learning methods (Table 3). All baseline models demonstrated high AUROC (area under the receiver operating characteristic curve) values (>90%), confirming their strong discriminative ability. Among them, XGBoost and LightGBM provided the best balance between sensitivity (45%) and precision (15–20%), which translated into the highest F1 scores and Average Precision (AP). In contrast, CatBoost achieved very high sensitivity (>70%) but at the cost of extremely low precision (<4%), while Random Forest yielded the highest specificity (99.95%) but also the lowest AP (<8%). The application of the Elkan & Noto method generally shifted the trade-off toward higher sensitivity but substantially reduced precision. This pattern was particularly pronounced for Random Forest, where sensitivity increased by over four times, but at the cost of a dramatic loss in precision and F-scores. Elkan & Noto often led to wider confidence intervals for sensitivity, especially in LightGBM (over four times), indicating reduced stability despite higher recall. The spy method produced more balanced results. In XGBoost and Random Forest, it improved sensitivity (by 71% and 14%, respectively), with a substantial reduction of variability, suggesting improved stability of the estimates. In CatBoost, however, spy slightly reduced sensitivity and further degraded precision, confirming the model’s limited suitability for this approach. LightGBM, on the other hand, showed improved sensitivity (by 31%) and narrower CI, but precision dropped by 54%. Notably, XGBoost predictions demonstrate the highest precision-sensitivity ratio, as evidenced by an AP of 24.53%, but the dispersion is very high (95% CI: 2.75–44.97).

**Figure 4:**
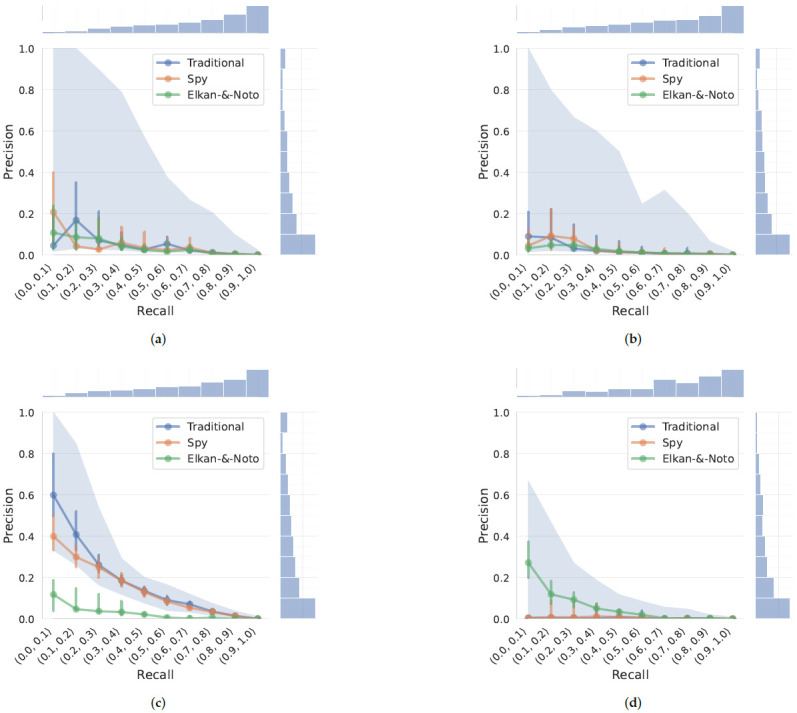
Precision–recall profiles for the four evaluated models: (**a**) XGBoost, (**b**) CatBoost, (**c**) LightGBM, and (**d**) Random Forest. Rather than standard continuous curves, these profiles illustrate the distribution of precision across specific 10% recall bins, allowing for a more robust assessment of performance. The vertical error bars represent the interquartile range (IQR; 25th to 75th percentile), showing the variability of precision around the median across all validation runs. The histograms on the top and right summarize how frequently different recall and precision values occurred. Additionally, the light blue shaded area highlights the 99% confidence interval of the traditional (baseline) model.

Moreover, we observed that standard AUPRC (area under the precision–recall curve) calculations using trapezoidal integration may substantially overestimate model performance due to the uneven distribution of observed precision–recall points, which in our case were highly concentrated in the high-recall/low-precision region (see marginal distributions in Figure 4). The default trapezoidal integration implicitly assumes linear interpolation between points, leading to inflated areas and potentially misleading conclusions, particularly in regions with sparse point density [46]. To address this bias, we used Average Precision (AP), which avoids linear interpolation by computing a weighted mean of precisions at each threshold (Table 3). For visual validation, we implemented a binning strategy (Figure 4) that provides direct insight into the precision–recall profile by showing the true data distribution undisturbed by interpolation. This representation confirms that LightGBM-based models are more stable and clearly outperform other candidates—a fact less evident with conventional PRC (precision–recall curve). The remaining models failed and PU methods yielded minor and inconsistent improvements, suggesting that they are unsuitable for this application. Although the Elkan and Noto approach led to a modest improvement in Random Forest, it overall did not achieve a competitive performance. Notably, the traditional, standalone LightGBM configuration consistently outperformed all PU-learning variants. To definitively rule out overfitting in our preferred LightGBM model, we visually inspected the training and validation learning loss curves (Supplement—Train and Valid Loss; Figure S1). The curves demonstrated stable convergence without the divergence characteristic of overfitting, confirming the effectiveness of our aggressive regularization and strict structural constraints. Consequently, subsequent analyses in this study focus exclusively on the LightGBM.

### 3.3. LightGBM—Model Interpretability and Clinical Insights

**Figure 5:**
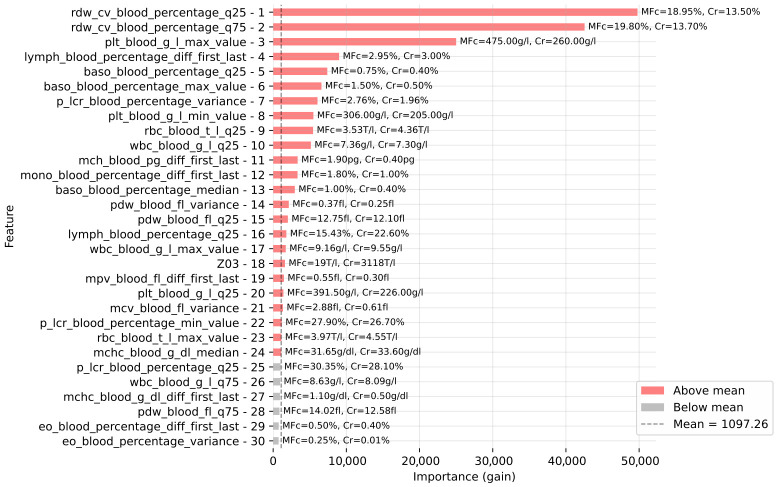
The 30 most important features based on gain for LightGBM. Each bar is labeled with the median feature value for the PMFc and Cr groups.

**Figure 6:**
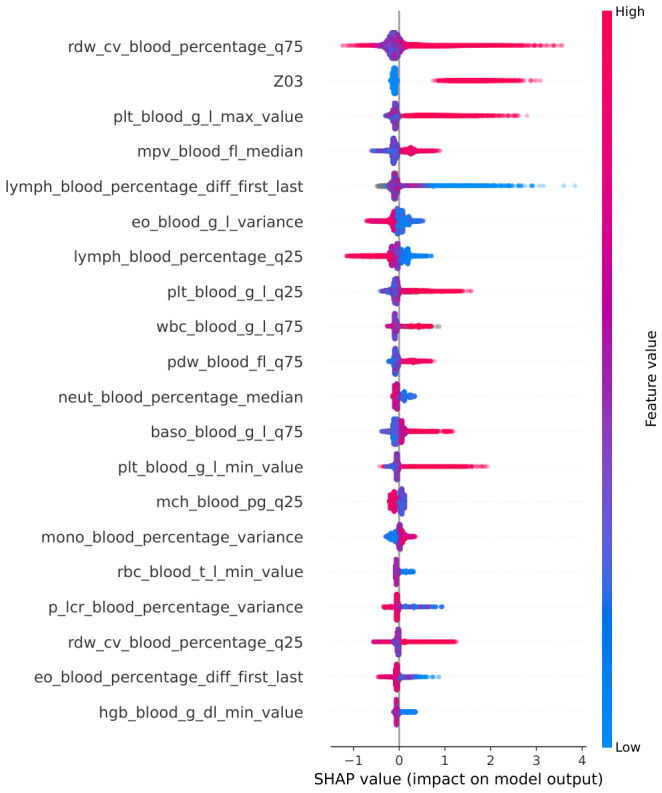
Summarization of SHAP values for the top 20 features.

To understand the best-performing model’s decision-making process, we conducted feature importance analysis using both gain and SHAP values (Figure 5 and Figure 6). Both methods revealed that the model’s predictions were primarily driven by CBC-derived features. The gain analysis showed that the most critical determinants were the distribution of RDW-CV and extremely high PLT values. Interestingly, CBC parameters connected with blood cells had notably lower importance. Among all white blood parameters, aggregates of lymphocytes and basophils were the most informative. The model also identified the ICD-10 code Z03 (diagnostic observation) as the most informative among all used ICD-10 codes and phenotypes. However, given its low total frequency in the cohort and the model’s overwhelming reliance on CBC parameters, the Z03 code acts as an auxiliary signal and does not replace the robust biological evidence, thereby mitigating concerns of significant label leakage.

SHAP (SHapley Additive exPlanations) value analysis confirmed the importance of high RDW-CV, extremely high PLT values, and changes in lymphocyte counts. However, the lower quantile of RDW-CV lost its significance but upper quantile deviations and high PLT levels frequently contributed towards positive predictions. In contrast, large changes in lymphocyte counts often were associated with predictions favoring the control group.

**Figure 7:**
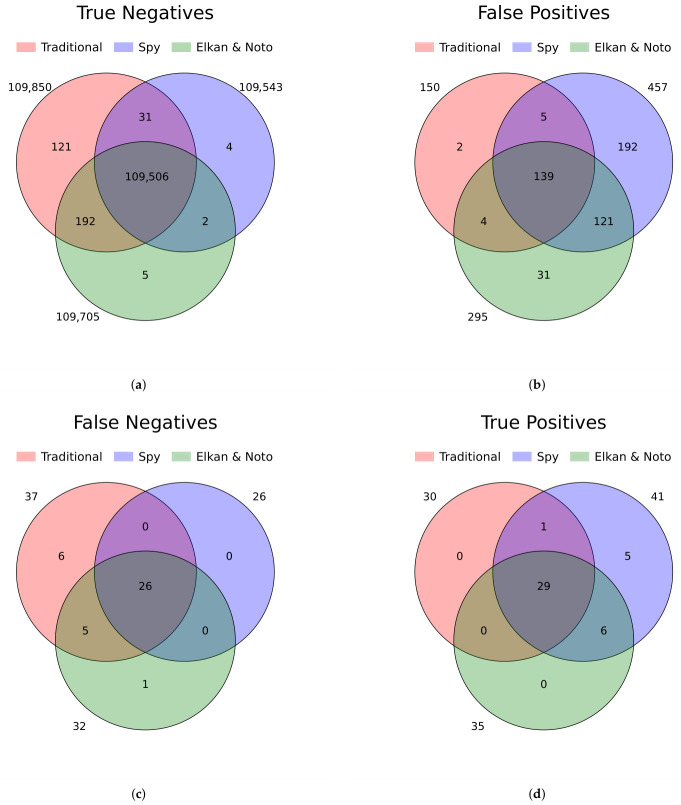
Venn diagrams illustrating number of patients with assigned specific label—the consensus between LightGBM model variants for each prediction category: (**a**) true negatives (TN), (**b**) false positives (FP), (**c**) false negatives (FN), and (**d**) true positives (TP). The numbers indicate how many patients were consistently classified into each category across models in more than half of the validation folds. These data are also presented as individual confusion matrices for each model variant in the Supplement—Confusion Matrices; Figure S2.

Venn diagrams combining the results of all LightGBM-based models revealed a high degree of concordance (Figure 7). Notably, the Spy and Elkan & Noto methods demonstrated higher sensitivity compared to the other approaches, but this comes at the cost of an increased false positive rate (FPR).

**Figure 8:**
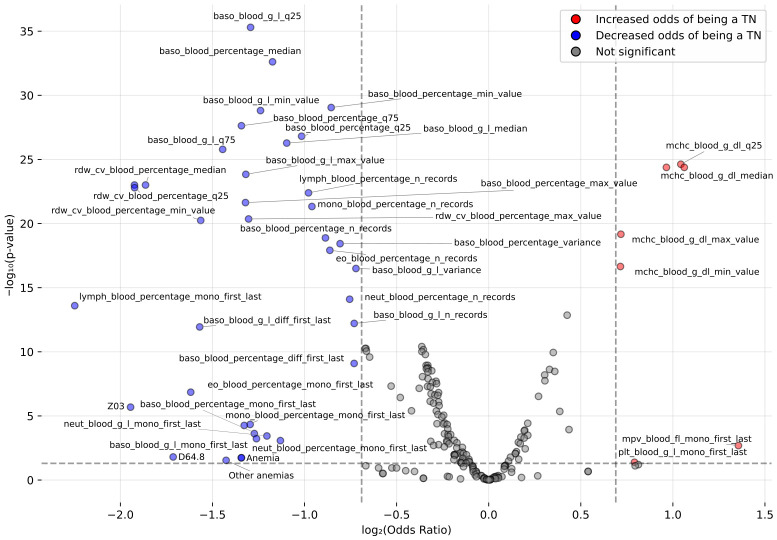
Volcano plot showing differences in features between Cr patients correctly (TN) and incorrectly (FP) by all models (in more than half of the validation folds).

Finally, we investigated the features of patients frequently misclassified by the models (listed on Figure 7) in comparison to correctly classified. Among positive cases (PMFc), no statistically significant differences were found between true positives (TP) and false negatives (FN), suggesting that missed positive cases shared similar profiles with those correctly identified. In contrast, among Cr patients, significant differences were observed between true negatives (TN) and false positives (FP) (Figure 8). The volcano plot (Figure 8) reflected a similar yet inverse pattern to Figure 3, suggesting that the FP (Cr) patients exhibited clinical characteristics closely resembling TP (PMFc) cases. This finding is critical: it suggests that these FP patients (n = 139) are highly likely to be undiagnosed or mislabeled PMF patients (hidden positives). This result directly supports the PU approach: model “errors” in this context are often not true classification failures, but the result of label bias and incompleteness inherent in EHR data.

The importance of CBC features identified by the algorithms did not fully align with clinical practice. Although PMF is a myeloproliferative disease with pathology primarily affecting the myeloid lineage (neutrophils, basophils, eosinophils, monocytes), both gain and SHAP-based analyses indicated a lower importance of features derived from these cell populations. Instead, feature-derived parameters such as RDW and PDW—generally regarded as nonspecific and of limited clinical utility—contributed substantially to the model (Figure 5 and Figure 6). Interestingly, among the white blood cell parameters, changes in lymphocyte counts were more strongly associated with the model’s performance than features derived from the myeloid lineage.

### 3.4. False Positive Patients Analyses

Due to limited access to physicians and the high number of false positives produced by PU (positive-unlabeled) approaches, we focused our manual clinical review on two groups: the intersection of false positives identified by all models (the consensus subset, Table 4) and all remaining false positives identified by the best-performing traditional LightGBM model (Table 5). A total of 150 patients were reviewed by two independent physician panels using a standardized framework based on clinical data (laboratory results, ICD-10 codes, and documentation). Labels covered both disease risk and diagnostic status. Discrepancies were resolved through consensus, with final decisions made by an experienced hematologist (>20 years of practice), ensuring consistency and reliability. Among patients in the consensus subset indicated by all models, five had a confirmed PMF diagnosis, none of whom had PMF-related ICD-10 codes in the EHR, while two showed high-risk and three medium-risk features. Six patients were suspected of PMF and were awaiting trephine biopsy, all with medium or high risk documented. PMF was excluded in 41 patients based on trephine biopsy results, although all had clinical suspicion of hematological malignancy; eight of these were assessed as medium or high-risk. Disease status was unknown in 72 patients, as no PMF diagnostics were performed; 10 of these were assessed as medium- or high-risk. Overall, 31 of 139 patients were classified as having medium or high risk of PMF. For all of them, the expert would be comfortable scheduling a follow-up visit to verify the suspicion based on documentation. Importantly, 51 patients underwent specialized hematological diagnostic procedures.

In addition, we verified the remaining patients identified by the traditional model (Table 5), outside the consensus subset. The review revealed that four patients classified as FPs lacked sufficient clinical data for a definitive exclusion or confirmation. The remaining seven patients were assessed as low risk.

## 4. Discussion

### 4.1. Methodological Innovations

In this study, we demonstrate the use of ensemble machine learning for identifying patients with PMF by leveraging limited information extracted from real-world EHRs (electronic health records). Our analysis centered on domain-driven construction of the training dataset and a comprehensive comparison of ensemble learning methods— specifically XGBoost, LightGBM, CatBoost, and Random Forest. These models were evaluated across diverse experimental setups, leveraging an AutoML (automated machine learning) framework to conduct exhaustive hyperparameter optimization and architecture search, alongside their adaptation to the Positive-Unlabeled (PU) learning paradigm. Notably, although we confirmed the inherently positive-unlabeled nature of the EHR-derived dataset, the integration of PU learning techniques did not translate into significant gains in precision-based metrics, such as Average Precision (AP). This lack of improvement is likely attributable to the extreme class imbalance and the scarcity of hidden positives typical of rare diseases; while these methods generally enhanced sensitivity, the effect remained inconsistent across different models, highlighting that the efficacy of PU-specific approaches is highly sensitive to the choice of the underlying base classifier. Experimental results demonstrated that LightGBM was superior in terms of the precision–recall ratio, likely due to its leaf-wise growth strategy, which effectively captures complex feature interactions and accommodates heterogeneity within sparse positive samples. In contrast, learners relying on target-based encoding, such as CatBoost or weight-of-evidence (WoE)-preprocessed Random Forests, were less effective in this specific clinical context.

Evaluation of models in this case presents unique challenges. The use of classical binary classification metrics (precision, sensitivity, specificity) for PU learning presents significant interpretability challenges due to the absence of fully verified true negative labels. In such settings, reported metrics should be viewed as approximations rather than absolute ground truth. While some literature [47] suggests using metrics concentrated on top-ranked cases, such as precision at *K* or recall at *K*, we argue that *K* must be explicitly anchored in clinical reality—reflecting either the expected prevalence of hidden positives or the operational capacity of the healthcare system (e.g., the throughput of diagnostic procedures). Without such deliberate justification, the choice of *K* remains arbitrary. Therefore, we prioritize AP as a comprehensive, threshold-agnostic measure that provides a more robust reflection of the model’s discriminative utility across the entire cohort. We observed greater variance in AP than in Receiver Operating Characteristic (ROC), underscoring the informational value of the precision–recall curve (PRC) for imbalanced classification tasks. However, we found that the common approach of calculating the area under the curve tends to overestimate performance due to implicit interpolation assumptions. To mitigate this, we adopted a binning strategy that aggregated the original data points within specific recall ranges, providing a more reliable and informative evaluation. Additionally, threshold-dependent metrics may fail to capture true model utility in practice. At the default operating point, the decision threshold effectively balances class weights inversely to their frequency, implicitly placing a higher cost on false negatives than on false positives. This trade-off may not be desirable from a clinical perspective, which underscores the need to contextualize these metrics by considering disease prevalence and defining clinically acceptable thresholds.

### 4.2. Comparison to Other Models of Hematological Malignancies

Our model achieved an AUROC of 96.63%, specificity of 99.84%, and sensitivity of 45.52% in an extremely imbalanced cohort (only 67 PMF cases among 110,000 controls). Despite this far greater class imbalance than in comparable studies, our model reached discriminative performance comparable to or exceeding that reported for other hematologic malignancies. For example, models predicting chronic myeloid leukemia (CML) from peripheral blood morphology achieved AUROC values of 87–92% [8], and large-scale myelodysplastic syndromes (MDS) risk prediction models reported an AUROC of 87.2% with sensitivity and specificity of 78.5% and 80.4%, respectively, [6]. Even deep learning systems distinguishing MDS from aplastic anemia (AA) or classifying Ph-negative myeloproliferative neoplasms (PV—polycythemia vera; ET—essential thrombocythemia; PMF—primary myelofibrosis) achieved AUROCs of 99% and 97.4%, respectively, but in small, relatively balanced datasets [5,48]. Similar to the AI-driven decision support systems for leukemia proposed by Turki et al. [3], our model focuses on leveraging basic laboratory parameters to bridge the diagnostic gap in non-specialist clinical environments. Our model’s exceptional specificity (99.84%) notably surpasses that of previous studies, while the lower sensitivity (45%) reflects both the extreme imbalance of our data and a threshold calibration.

Importantly, our evaluation framework addresses the limitations of AUROC under class imbalance by incorporating precision-based analysis (AP ≈ 21%), which substantially exceeds that reported in the study [6] related to other rare disease—myelodysplastic syndrome (≈8%). Most prior studies omitted precision-based metrics, relying on uncalibrated, threshold-dependent measures such as sensitivity and specificity. We highlight that PR-based evaluation should be standard for imbalanced data, and that threshold-dependent metrics must be properly calibrated to ensure meaningful and comparable results. Additionally, we report confidence intervals for all key metrics—a practice often neglected but essential for robust and reproducible assessment, especially when the sample size is small.

Among machine learning approaches applied to hematologic data, XGBoost has been the most frequently chosen model, owing to its strong ability to capture complex nonlinear relationships and robustness to missing values [5,6,8,48]. In contrast, artificial neural networks (ANNs) and logistic regression have typically been used for comparison rather than as primary predictive tools [6,8]. In our study, however, LightGBM outperformed XGBoost, suggesting that LightGBM should also be systematically included in future benchmarking efforts, particularly in large-scale and highly imbalanced clinical datasets.

### 4.3. Interpretability of Model Findings and Their Clinical Relevance

Our study presents a machine learning framework for the identification of PMF patients using routinely collected electronic health records (EHR) data. The results demonstrate that PMF patients can be reliably distinguished based on subtle yet progressive alterations in their hematological parameters and features extracted from clinical documentation. Notably, the features prioritized by our models differ from those traditionally emphasized in standard clinical practice; while previous studies have reported the prognostic significance of parameters such as RDW, PDW, and basophil count for disease progression or risk of transformation, these variables are not routinely utilized to identify patients with suspected PMF. Importantly, the model did not rely on absolute laboratory values but on aggregated representations derived from comparisons across multiple measurements, reflecting temporal trends and deviations from individual baselines. This contrasts with studies based on pre-defined clinical cohorts, which may fail to detect early or subclinical indicators of disease. Collectively, these findings highlight the potential of data-driven approaches, such as ours, to enable recognition of PMF in routine care settings, ultimately improving patient outcomes through timelier diagnostic intervention.

A notable observation is that classical myeloid parameters appeared less influential in the model compared to more nonspecific indices such as RDW or platelet-related measures. This finding warrants careful interpretation. From a biological perspective, it may reflect the early and systemic nature of PMF, in which subtle dysregulation of hematopoiesis manifests initially through nonspecific variability rather than overt lineage-specific abnormalities. From a data perspective, routinely collected EHR data are subject to variability in measurement frequency, completeness, and clinical context, which may attenuate the signal of less frequently assessed or more variable parameters such as differential counts. Finally, from a modeling standpoint, tree-based ensemble methods may preferentially select features that capture stable, longitudinal patterns and maximize predictive performance, even if these features are less specific from a pathophysiological perspective. Therefore, the observed feature importance likely represents a combination of underlying disease biology, real-world data constraints, and algorithmic behavior, rather than a direct contradiction of established clinical knowledge.

It is crucial to acknowledge that PMF is a highly heterogeneous disease, characterized by distinct phenotypes ranging from cytopenic to proliferative manifestations [49]. While our model utilizes a binary classification (PMF vs. control) for methodological purposes, this clinical diversity may influence the sensitivity of automated screening across different disease stages.

### 4.4. Changes in Interpretation of False Positive

Our analysis of false-positive cases yields an important insight: patients classified as false positives should not be readily dismissed as true negatives when working with real-world EHR data. Manual verification revealed that while the FP group is not dominated by “hidden positives”, it contains critical unlabeled positive patients that are difficult to identify in such an imbalanced setting (prevalence ≈ 0.061%). We identified individuals who were ultimately diagnosed with PMF after the study’s data extraction period or whose diagnosis was not captured due to missing or incorrect ICD-10 coding. The presence of even a small number of such cases (n = 5 confirmed PMF among the reviewed subset) in such a vast population provides strong empirical evidence that EHR datasets are inherently PU in nature. Achieving perfectly accurate labels is virtually impossible, even with extensive manual review. The model’s ability to surface these cases from over 110,000 individuals is a significant finding.

Although the proportion of true PMF cases within the false-positive group was relatively low, a substantial number of these patients—nearly 37%—had already undergone specialized hematological diagnostic procedures, including bone marrow biopsies, in clinical practice. Although these biopsies ultimately excluded PMF in 41 cases, the fact that these patients were referred for such invasive investigations by clinicians confirms that the model accurately identifies individuals with high-risk clinical profiles that are difficult to distinguish from PMF without specialist intervention. As expected, the majority were ultimately diagnosed with some form of myeloproliferative neoplasm. This suggests that focusing exclusively on PMF diagnosis may not be optimal given the constraints of the available data. Instead, identifying broader disease categories, such as myeloproliferative disorders as a whole, may represent a more suitable and clinically meaningful objective.

Furthermore, our results highlight the potential of these algorithms to identify patients who are overlooked by standard clinical workflows. Analysis of the overlap in false positives across different models indicates that the models consistently capture phenotypic patterns closely resembling those of patients with PMF. This observation, combined with evidence that the algorithms process information differently from human clinicians, suggests that approaches such as ours can effectively surface patients who are missed in routine clinical practice.

### 4.5. Clinical Application and Screening Framework

To translate these findings into clinical practice, we propose a framework where the model acts as a preliminary prioritization system within large-scale EHR populations. Given the extremely low prevalence of PMF (≈0.061%) in our study population, the model achieved over 340-fold enrichment (20.81/0.061) of cases, effectively surfacing high-risk individuals who would otherwise remain hidden in the vast volume of routine data. Our algorithm remains applicable in non-specialist settings like primary care, serving as an early screening tool to facilitate timely referrals rather than replacing definitive diagnostic standards. To estimate the workload for the health system, we assumed 15 min of physicians time per patient, similar to a standard outpatient visit, reflecting the time needed to review EHR data. The median of flagged patients per hospital was 15, corresponding to 3.75 h of additional doctor’s time.

We propose a two-stage longitudinal workflow where the algorithm serves as an initial filter to identify a manageable subset (e.g., the top 1% of the population). This focused cohort is then subjected to manual clinical review by hematologists. The practical utility of this approach is supported by our manual review findings: 37% of flagged patients had already triggered clinical suspicion leading to specialized procedures, and 22% were independently classified by clinicians as carrying medium-to-high risk. These results indicate that the model’s “errors” are often clinically relevant manifestations that warrant investigation, rather than simple noise. This screening framework converts modest statistical precision into a sustainable diagnostic tool, ensuring that specialist resources are directed toward the most critical cases within real-world resource constraints.

### 4.6. Limitations and Future Directions

The generalizability of our findings is subject to certain limitations. The positive class comprised 67 patients, all of whom were included in the repeated validation process to maximize statistical reliability, and they originated from just 3 of the 10 participating hospitals. This raises concerns regarding potential overfitting to positive samples and hospital-specific biases. While repeated cross-validation is a robust technique, we acknowledge that using global hyperparameter tuning may introduce an optimistic bias; however, this risk was proactively mitigated through aggressive regularization, strict constraints on tree depth, and rigorous stability analysis across all repetitions to prevent learning memorized patterns. Importantly, the use of an age- and gender-matched control cohort was intended to reduce confounding and increase task difficulty, thereby preventing the model from relying on simple demographic signals; however, this design also limits the direct translation of precision estimates to real-world settings, particularly given differences between hospital-based EHR populations and the general population. Furthermore, the algorithm predicts the risk of an individual already having a diagnosis of PMF based on their record, rather than providing clinical risk stratification for the disease itself. Its role is intended as a screening tool to prioritize patients for hematological evaluation. Additionally, the absence of cross-hospital patient identifiers introduces an unquantifiable risk that a patient may appear multiple times under different IDs or appear in both the Cr and PMFc groups. Future work should focus on validating the model on a representative general population, quantifying generalizability across different hospital settings, and calibrating the decision threshold to ensure alignment with real-world clinical applicability. Furthermore, the inclusion of specific administrative ICD-10 codes, such as Z03, may introduce bias reflecting institutional or individual clinician practices. The impact of such codes on predictions must be verified in subsequent studies. While our observations confirm the PU nature of clinical datasets, integrating PU methods did not yield practical benefits—the increase in sensitivity was accompanied by a significant decline in precision, generating an unmanageable screening burden that surpasses our operational limits. Nevertheless, we believe that this paradigm should be extensively tested in future studies; the disconnect between clinical reality and model performance suggests a need for deeper investigation into how PU learning can be better adapted to the extreme imbalances inherent in EHR data.

## 5. Conclusions

We compared ensemble models and PU learning approaches to address heterogeneity, data incompleteness, and underrepresentation of the positive class. To ensure practical utility within resource-constrained clinical environments, we emphasized stability and precision, favoring a manageable number of high-fidelity predictions. Our framework is intended as a preliminary screening tool to identify high-risk individuals for hematological evaluation, not a standalone diagnostic instrument. Our analysis of multi-center data across 10 Polish hospitals established LightGBM as the optimal model, achieving the most stable high precision (AP of 20.83%, precision of 14.72%) with a sensitivity of 45.52%. The model effectively extracted diagnostic value from interactions between nonspecific parameters—RDW and PLT—ensuring predictive power that was not compromised by underlying data sparsity.

Notably, while PU methods can enhance sensitivity, this gain comes at a substantial cost to precision, resulting in a volume of predictions that exceeds the operational capacity of real-world screening workflows. Nevertheless, our analysis revealed that some unlabeled patients identified as high-risk actually possessed confirmed diagnoses or clinical profiles closely resembling confirmed cases. This offers compelling empirical evidence that clinical EHR datasets are fundamentally PU in nature. It also demonstrates that, despite rigorous data curation, achieving perfectly verified negative labels remains impossible in real-world settings, thus validating the theoretical foundation for PU learning. Furthermore, although myelofibrosis is a heterogeneous disease, the consistent overlap of true positives detected by both supervised and PU approaches highlights their shared ability to identify core PMF phenotypes. Ultimately, by processing information differently from clinicians, this methodology shows significant potential to bridge the diagnostic gap and surface high-risk patients missed during routine clinical care.

These findings underscore why PU learning deserves further investigation in future algorithm development for EHR data; while it is not a replacement for established supervised methods, we recommend that PU-aware algorithms should be extensively tested in clinical domains where inherent label incompleteness is inevitable. This is especially relevant in screening scenarios where sensitivity is preferred over precision and the cost of false negatives substantially exceeds that of false positives.

## Figures and Tables

**Table 1:** The number of patients from several levels of selection.

Parameter	Value
Total number of PMF patients identified	448
Number of PMFc cases	233
Number of PMFc with CBC	171
Number of PMFc with CBC prior to the diagnosis	67
Total number of Cr	3,360,236
Number of Cr with CBC results	1,092,913
Cr sampling stratified by age and gender of PMFc	110,000

**Table 2:** Patient demographics and clinical characteristics of the cohorts. CBC values were summarized as the median per patient, and reported values correspond to the median within Cr and PMFc groups.

Category	Characteristic	Predictor	Cr (n = 110,000)	PMFc (n = 67)	# *p* Value
Demographic	Age	TRUE	63 (28, 86)	69 (40, 88)	NS
	Sex—Female	TRUE	51.65% (56,820 patients)	53.73% (36 patients)	NS
	Sex—Male	TRUE	48.35% (53,180 patients)	46.27% (31 patients)	NS
Diagnosis	ICD-10 (at least one code)	TRUE	avg. 0.25 per person	avg. 0.77 per person	***
	Phenotypes (optional)	TRUE	avg. 0.19 per person	avg. 0.56 per person	***
Visits	number of visits	FALSE	3 (1, 27)	5 (1, 40)	***
	time span	FALSE	avg. > 1 mth.	avg. > 4 mths.	***
	visits per year	FALSE	avg. > 1 per year	avg. > 2 per year	***
CBC	RBC_blood_t_l	TRUE	4.41 (2.88, 5.51)	3.67 (2.29, 6.55)	***
	HGB_blood_g_dl	TRUE	13.30 (8.60, 16.60)	10.20 (7.38, 15.12)	***
	HCT_blood_percentage (optional)	TRUE	39.50 (26.20, 48.70)	33.60 (23.05, 47.88)	***
	MCV_blood_fl (optional)	TRUE	89.50 (77.10, 102.00)	91.55 (74.00, 116.14)	NS
	MCH_blood_pg (optional)	TRUE	30.20 (24.40, 34.50)	29.02 (22.08, 38.11)	*
	MCHC_blood_g_dl (optional)	TRUE	33.60 (30.80, 35.90)	31.65 (28.50, 34.00)	***
	RDW_CV_blood_percentage (optional)	TRUE	13.60 (11.90, 19.90)	19.45 (13.38, 26.37)	***
	WBC_blood_g_l	TRUE	7.67 (3.55, 19.33)	8.00 (2.75, 72.95)	NS
	NEUT_blood_g_l	TRUE	4.68 (1.58, 15.48)	5.53 (1.14, 65.16)	NS
	LYMPH_blood_g_l	TRUE	1.67 (0.50, 3.82)	1.46 (0.70, 5.03)	NS
	MONO_blood_g_l	TRUE	0.60 (0.22, 1.55)	0.72 (0.22, 2.88)	NS
	BASO_blood_g_l	TRUE	0.03 (0.00, 0.10)	0.08 (0.00, 0.76)	***
	EO_blood_g_l	TRUE	0.11 (0.00, 0.54)	0.12 (0.00, 2.75)	NS
	NEUT_blood_percentage	TRUE	64.30 (35.01, 89.00)	64.90 (41.18, 86.91)	NS
	LYMPH_blood_percentage	TRUE	23.60 (4.90, 49.15)	16.75 (3.26, 41.29)	*
CBC	MONO_blood_percentage	TRUE	8.10 (3.00, 15.40)	7.60 (2.40, 19.14)	NS
	EO_blood_percentage	TRUE	1.50 (0.00, 6.90)	1.80 (0.06, 5.28)	NS
	BASO_blood_percentage	TRUE	0.40 (0.00, 1.30)	1.00 (0.00, 2.49)	***
	PLT_blood_g_l	TRUE	233.00 (73.50, 468.00)	416.00 (33.00, 1418.00)	***
	PDW_blood_fl (optional)	TRUE	12.30 (9.10, 18.40)	13.30 (9.68, 25.40)	*
	MPV_blood_fl (optional)	TRUE	10.25 (7.40, 12.65)	11.05 (8.95, 13.04)	***
	PLCR_blood_percentage (optional)	TRUE	28.80 (15.70, 46.60)	33.60 (19.45, 48.71)	**

# Continuous variables: Median (min, max); Categorical variables: Frequency (% and count). Adjusted *p*-value: * *p* < 0.05, ** *p* < 0.01, *** *p* < 0.001,NS = not significant. RBC—Red BloodCells (Erythrocytes);HGB—Hemoglobin;HCT—Hematocrit;MCV—Mean Corpuscular Volume;MCH—Mean Corpuscular Hemoglobin;MCHC—Mean Corpuscular Hemoglobin Concentration; RDW—Red Cell DistributionWidth; WBC—White Blood Cells (Leukocytes); NEUT—Neutrophils; LYMPH—Lymphocytes; MONO—Monocytes; EO—Eosinophils; BASO—Basophils; PLT—Platelets (Thrombocytes); PDW—Platelet DistributionWidth;MPV—Mean Platelet Volume; PLCR—Platelet Large Cell Ratio.

**Table 3:** Comparison of model characteristics based on 10 repetitions of 10-fold cross-validation. For each model, the table reports the median and 95% confidence interval across all 100 validation sets. The best results are bolded, and the second-best are underlined.

Model	AUROC [%]	AP [%]	Specificity [%]	Sensitivity [%]	Precision [%]	F1 [%]
XGBoost	98.03 (92.47–99.69)	**24.53 (2.75–44.97)**	99.93 (99.89–99.96)	32.26 (6.33–54.57)	**20.34 (4.72–41.64)**	**26.07 (5.24–44.16)**
XGBoost-Elkan-&-Noto	97.44 (93.84–99.63)	14.08 (2.29–25.39)	99.79 (99.63–99.85)	47.14 (13.86–69.64)	11.40 (3.71–17.84)	19.31 (5.78–26.53)
XGBoost-spy	97.60 (97.59–98.52)	17.76 (2.65–39.11)	99.68 (99.59–99.75)	55.22 (45.45–57.87)	9.43 (7.73–10.09)	16.04 (13.60–17.19)
CatBoost	**98.36 (93.09–99.54)**	16.05 (2.02–42.60)	98.44 (98.38–98.57)	79.29 (43.33–89.68)	3.13 (1.44–3.63)	6.03 (2.80–6.97)
CatBoost-Elkan-&-Noto	97.98 (92.55–99.37)	5.19 (1.31–10.18)	97.61 (97.53–97.84)	**82.86 (51.12–97.29)**	2.16 (1.15–2.59)	4.20 (2.25–5.04)
CatBoost-spy	97.47 (96.80–98.29)	13.96 (2.01–39.01)	97.72 (97.50–97.89)	77.61 (73.47–80.60)	2.03 (1.84–2.13)	3.96 (3.60–4.14)
LightGBM	96.46 (95.99–98.22)	20.83 (19.18–24.35)	99.84 (99.82–99.85)	45.52 (39.48–52.72)	14.72 (13.80–17.01)	22.31 (20.44–25.25)
LightGBM-Elkan-&-Noto	97.72 (85.42–99.58)	12.09 (2.19–21.84)	99.64 (99.57–99.71)	58.57 (20.58–75.86)	9.42 (3.12–11.71)	16.38 (5.40–20.15)
LightGBM-spy	97.39 (96.72–98.49)	16.21 (13.80–21.20)	99.51 (99.41–99.59)	59.70 (57.05–65.67)	6.78 (5.97–8.22)	12.21 (10.84–14.47)
RandomForest	93.81 (78.18–97.78)	7.14 (0.34–17.71)	**99.95 (99.93–99.97)**	15.00 (0.00–24.05)	13.18 (0.00–30.95)	14.82 (0.00–26.45)
RandomForest-Elkan-&-Noto	94.40 (84.22–98.99)	9.92 (5.11–29.41)	98.10 (97.92–98.27)	64.50 (56.67–89.49)	1.69 (1.52–2.30)	3.28 (2.97–4.48)
RandomForest-spy	90.93 (89.17–95.36)	7.68 (0.44–21.46)	99.94 (99.93–99.95)	17.16 (13.77–23.54)	14.17 (11.99–16.94)	15.43 (13.18–19.70)

**Table 4:** Manual clinical review of consensus subset false positive predictions, presenting EHR-based disease risk and assigned clinical diagnoses.

Risk	Diagnostic Status			
	**Confirmed**	**Excluded**	**Unknown**	**Suspected**
Low	0	37	62	0
Medium	3	8	8	6
High	2	1	2	1
No risk	0	0	3	0
No data	0	0	5	1

**Table 5:** Manual clinical review of the remaining LightGBM false positives (excluding consensus subset), presenting EHR-based disease risk and assigned clinical diagnoses.

Risk	Diagnostic Status			
	**Confirmed**	**Excluded**	**Unknown**	**Suspected**
Low	0	0	7	0
Medium	0	0	0	0
High	0	0	0	0
No risk	0	0	0	0
No data	0	0	4	0

## Data Availability

The data presented in this study are available on request from the corresponding author. The data are not publicly available due to personal data and privacy protection.

## Supplementary Materials

The following supporting information can be downloaded at: https://www.mdpi.com/article/10.3390/cancers18101618/s1, Supplementary Table S1: Hyperparameter tuning settings; Supplementary Table S2: Distribution statistics and feature frequencies in the PMFc and Cr groups, with univariate comparison *p*-values (FDR-adjusted); Supplement—Details of PU methods: Details of PU methods; Supplement—Train and Valid Loss. Figure S1: Averaged loss function of LightGBM across training and validation folds; Supplement—Confusion Matrices. Figure S2: Confusion matrices for the evaluated variants of the LightGBM model;
