# Supplementary material for: Leveraging Ensemble Machine Learning Models for the Detection of Primary Myelofibrosis in Electronic Health Records
Source: Cancers (Basel). 2026 May 16;18(10):1618. doi: 10.3390/cancers18101618 (PMC13204187; doi:10.3390/cancers18101618)
Supplement: Supplementary file 1 [file cancers-18-01618-s001.zip › Supplement - Confusion Matrices.pdf]

Supplement - Confusion Matrices

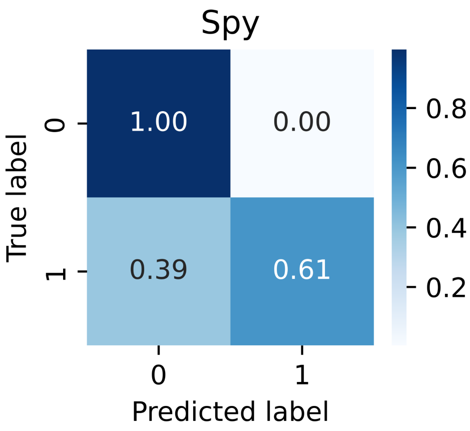

(a)

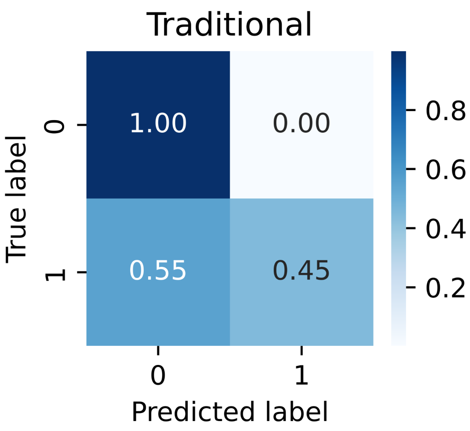

(b)

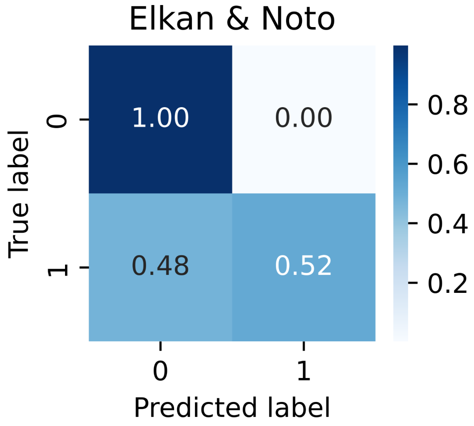

(c)

Figure S2. Confusion matrices for the evaluated variants of the LightGBM model: **(a)** standard supervised approach - traditional, **(b)** Elkan and Noto method, and **(c)** Spy technique. Each matrix summarizes the classification performance in terms of true positives, false positives, true negatives, and false negatives based on the number of patients consistently assigned to a given category in more than half of the validation splits. Figure 7 in the main manuscript presents the consensus between models.
