# Supplementary material for: Leveraging Ensemble Machine Learning Models for the Detection of Primary Myelofibrosis in Electronic Health Records
Source: Cancers (Basel). 2026 May 16;18(10):1618. doi: 10.3390/cancers18101618 (PMC13204187; doi:10.3390/cancers18101618)
Supplement: Supplementary file 1 [file cancers-18-01618-s001.zip › Supplement - Details of PU methods.pdf]

Supplement - Details of PU methods

Positive-Unlabeled (PU) learning is a branch of semi-supervised learning where the training data consists of a set of labeled positive examples and a set of unlabeled examples (which may contain both hidden positives and true negatives). These methods aim to leverage the inherent clustering of positive cases in the feature space, allowing for more robust boundary estimation when explicit negative labels are missing. In this study, we selected two of the most widely recognized strategies for adapting conventional binary classification models to the PU setting. All models mentioned in the manuscript were adapted using these two distinct strategies: the Elkan & Noto method [1] and the Spy method [2].

Elkan & Noto Method

The Elkan & Noto method [1] is based on the assumption that labeled positive samples are selected completely at random from the set of all positive samples (the SCAR assumption - Selected Completely At Random). The process involves the following steps:

1. **Calibration Set:** A fraction (10% in our case) of the labeled positive samples is held out as a validation set.
2. **Initial Classification:** A classifier is trained on the remaining data, where all labeled samples are treated as positive (P) and all unlabeled samples (U) are treated as negative. This classifier predicts $P(s = 1|x)$.
3. **Estimating c:** To estimate c directly from PU data, as suggested by Elkan and Noto, the initial classifier is applied to the held-out positive samples to estimate the constant $c = P(s = 1|y = 1)$, which represents the probability that a positive sample is labeled. The label frequency c is estimated as the average predicted probability that the labeled validation examples belong to the labeled class.
4. **Probability Correction (Postprocessing):** For any new sample, the initial classifier provides a probability $f(x) = P(s = 1|x)$. The true probability of being positive, $P(y = 1|x)$, is then estimated using the correction formula: $P(y = 1|x) = f(x)/c$. This adjusts the scores to account for the fact that the "negatives" in the training set were actually a mixture of positives and negatives.

Spy Method

The Spy method [2] is a two-step technique designed to identify "reliable negatives" within the unlabeled set. The process involves purposefully "infecting" the unlabeled data to create a threshold for filtering.

Our implementation follows the original pipeline:

1. **Inserting Spies (Deployment):** A small percentage (10% in our case) of labeled positive samples are randomly selected and moved to the unlabeled set—these are referred to as "spies". The algorithm knows they are truly positive, even though they are now hidden among the unlabeled data.
2. **First-Step Training:** An initial classifier is trained by treating the remaining labeled positives as the positive class and the entire augmented unlabeled set (including the spies) as the negative class.
3. **Thresholding:** After training, the classifier is used to predict posterior probabilities for all samples in the unlabeled set. Since the spies are known to be positive, their predicted scores serve as a reference for positive-like behavior within the unlabeled pool. A threshold is determined such that a desired fraction of spies (to account for potential noise, in our case 40%) have scores above it.
4. **Identifying Reliable Negatives:** Any unlabeled example that has a posterior probability lower than that of the lowest-scoring spy (or our adjusted threshold) is classified as a "reliable negative" (RN).
5. **Final Training:** Once the reliable negatives are identified, the "spies" are returned to the positive set, and a final classifier is trained using the original labeled positive set and the newly identified RN set. In our implementation, these reliable negatives, along with the remaining positives, were used to train the final classifier.

References

[1] Elkan, C., & Noto, K. (2008, August). Learning classifiers from only positive and unlabeled data. In Proceedings of the 14th ACM SIGKDD international conference on Knowledge discovery and data mining (pp. 213-220).

[2] Liu, B., Lee, W. S., Yu, P. S., & Li, X. (2002, July). Partially supervised classification of text documents. In ICML (Vol. 2, No. 485, pp. 387-394).
