# Supplementary material for: Leveraging Ensemble Machine Learning Models for the Detection of Primary Myelofibrosis in Electronic Health Records
Source: Cancers (Basel). 2026 May 16;18(10):1618. doi: 10.3390/cancers18101618 (PMC13204187; doi:10.3390/cancers18101618)
Supplement: Supplementary file 1 [file cancers-18-01618-s001.zip › Supplement - Train and Valid Loss.pdf]

Supplement - Train and Valid Loss

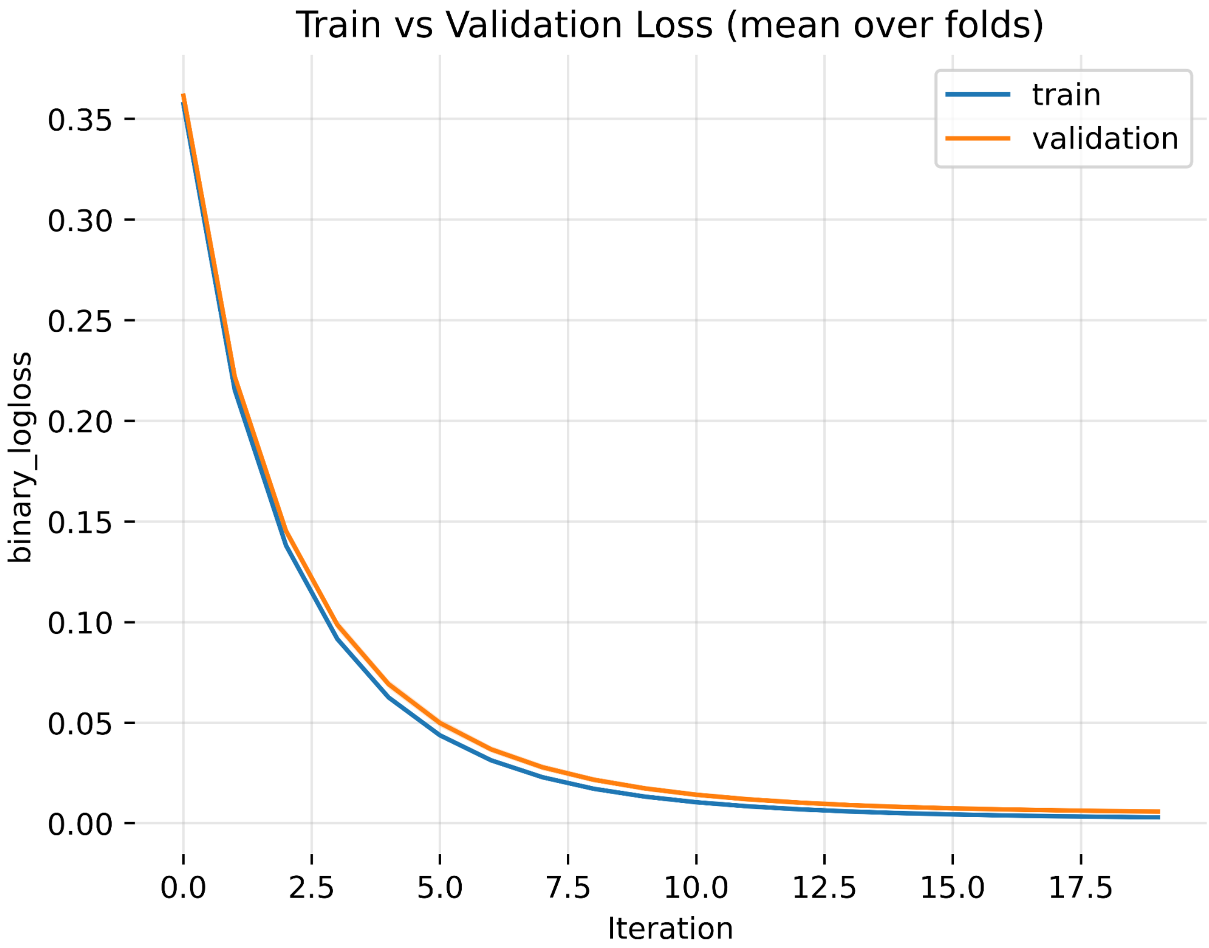

Figure S1: Averaged loss function of LightGBM across training and validation folds
