# Supplementary material for: Leveraging Ensemble Machine Learning Models for the Detection of Primary Myelofibrosis in Electronic Health Records
Source: Cancers (Basel). 2026 May 16;18(10):1618. doi: 10.3390/cancers18101618 (PMC13204187; doi:10.3390/cancers18101618)
Supplement: Supplementary file 1 [file cancers-18-01618-s001.zip › Supplementary_Table_S1.pdf]

Supplementary Table S1. Hyperparameter tuning settings

Algorithm	Hyperparameter	Options	Lower bound	Upper bound	Sampling
XGBoost / XGBoostPU	n_estimators	-	2	20	uniform
	max_depth	-	2	10	uniform
	min_child_weight	-	2	20	uniform
	min_split_loss	-	0.1	1	log-uniform
	booster	dart	-	-	fixed
	learning_rate	-	0.01	1	log-uniform
	max_delta_step	-	1	10	uniform
	eval_metric	AUCPR	-	-	fixed
	class_weight (scale_pos_weight)	balanced	-	-	fixed
	reg_alpha	-	1E-08	10	uniform
	reg_lambda	-	1E-08	10	uniform
	subsample	-	0.01	1	uniform
	colsample_bytree	-	0.3	1	uniform
	colsample_bylevel	-	0.3	1	uniform
	n_jobs	-1	-	-	fixed
CatBoost / CatBoostPU	n_estimators (iterations)	-	2	20	uniform
	max_depth (depth)	-	2	10	uniform
	learning_rate	-	0.01	1	log-uniform
	bagging_temperature	-	0	1	uniform
	border_count	-	2	10	uniform
	class_weight (scale_pos_weight)	balanced	-	-	fixed
	l2_leaf_reg	-	1E-08	10	log-uniform
	subsample	-	0.8	1	uniform
	leaf_estimation_iterations	-	1	10	uniform
	random_strength	-	1E-08	10	log-uniform
	n_jobs	-1	-	-	fixed
LightGBM / LightGBMPU	n_estimators	-	2	20	uniform
	max_depth	-	2	10	uniform
	num_leaves	-	10	150	uniform
	min_child_samples	-	2	100	uniform
	boosting_type	gbdt<br>dart<br>goss	-	-	uniform
	learning_rate	-	0.01	10	log-uniform
	max_bin	-	2	10	
	class_weight	balanced	-	-	fixed
	reg_alpha	-	1E-08	10	log-uniform
	reg_lambda	-	1E-08	10	log-uniform
	subsample	-	0.8	1	uniform
	colsample_bytree	-	0.8	1	uniform
	feature_fraction	-	0.8	1	uniform
	bagging_fraction	-	0.8	1	uniform
	n_jobs	-1	-	-	fixed
RandomForest / RandomForestPU	n_estimators	-	2	20	uniform
	max_depth	-	2	10	uniform
	min_samples_split	-	2	20	uniform
	min_samples_leaf	-	2	20	uniform
	max_features	sqrt<br>log2<br>None	-	-	uniform
	class_weight	balanced	-	-	fixed
	criterion	gini<br>entropy	-	-	uniform
	bootstrap	True<br>False	-	-	uniform
	n_jobs	-1	-	-	fixed
