# Supplementary material for: Leveraging Ensemble Machine Learning Models for the Detection of Primary Myelofibrosis in Electronic Health Records
Source: Cancers (Basel). 2026 May 16;18(10):1618. doi: 10.3390/cancers18101618 (PMC13204187; doi:10.3390/cancers18101618)
Supplement: Supplementary file 1 [file cancers-18-01618-s001.zip › Supplementary_Table_S2.pdf]

Supplementary Table S2. Distribution statistics and feature frequencies in the PMFc and Cr groups, with univariate comparison p-values (FDR-adjusted)							
**Origin**	**Feature**	**Cr Support**	**PMFc Support**	**p-value**	**Odds ratio (95% CI)**	**Cr Median (95% CI)**	**PMFc Median (95% CI)**
Continues laboratory	baso_blood_percentage_median	88679 (81%)	66 (99%)	***	1.7 (1.5, 1.9)	0.40 (0.00, 1.30)	1.00 (0.00, 2.49)
	baso_blood_percentage_q75	88679 (81%)	66 (99%)	***	1.8 (1.6, 2.1)	0.40 (0.00, 1.40)	1.06 (0.16, 3.67)
	baso_blood_g_l_q75	96631 (88%)	66 (99%)	***	1.7 (1.5, 1.9)	0.03 (0.00, 0.10)	0.09 (0.01, 1.02)
	mchc_blood_g_dl_q25	109598 (100%)	67 (100%)	***	0.5 (0.4, 0.6)	33.40 (30.50, 35.80)	31.30 (28.10, 33.74)
	rdw_cv_blood_percentage_median	104093 (95%)	67 (100%)	***	2.5 (2.0, 3.1)	13.60 (11.90, 19.90)	19.45 (13.38, 26.37)
	rdw_cv_blood_percentage_q25	104093 (95%)	67 (100%)	***	2.4 (1.9, 3.0)	13.50 (11.80, 19.30)	18.95 (13.22, 25.62)
	baso_blood_g_l_q25	96631 (88%)	66 (99%)	***	1.7 (1.5, 1.9)	0.03 (0.00, 0.10)	0.07 (0.00, 0.65)
	rdw_cv_blood_percentage_q75	104093 (95%)	67 (100%)	***	2.8 (2.2, 3.6)	13.70 (11.90, 20.70)	19.80 (13.79, 26.98)
	baso_blood_percentage_max_value	54723 (50%)	59 (88%)	***	1.8 (1.6, 2.1)	0.50 (0.00, 2.10)	1.50 (0.45, 8.36)
	mchc_blood_g_dl_median	109598 (100%)	67 (100%)	***	0.6 (0.5, 0.6)	33.60 (30.80, 35.90)	31.65 (28.50, 34.00)
	mchc_blood_g_dl_q75	109598 (100%)	67 (100%)	***	0.6 (0.5, 0.7)	33.72 (31.00, 36.02)	32.02 (28.79, 34.53)
	rdw_cv_blood_percentage_max_value	64716 (59%)	60 (90%)	***	2.2 (1.8, 2.7)	14.20 (11.90, 23.10)	20.80 (13.89, 29.67)
	baso_blood_percentage_q25	88679 (81%)	66 (99%)	***	1.5 (1.3, 1.7)	0.40 (0.00, 1.20)	0.75 (0.00, 2.24)
	rdw_cv_blood_percentage_min_value	64716 (59%)	60 (90%)	***	2.1 (1.7, 2.5)	13.30 (11.70, 19.00)	17.20 (12.89, 24.06)
	baso_blood_g_l_max_value	59405 (54%)	59 (88%)	***	1.7 (1.5, 1.9)	0.04 (0.00, 0.17)	0.12 (0.01, 0.90)
	baso_blood_percentage_min_value	54723 (50%)	59 (88%)	***	1.5 (1.3, 1.7)	0.30 (0.00, 1.10)	0.70 (0.00, 1.95)
	baso_blood_g_l_median	96631 (88%)	66 (99%)	***	1.4 (1.3, 1.6)	0.03 (0.00, 0.10)	0.08 (0.00, 0.76)
	baso_blood_percentage_variance	54723 (50%)	59 (88%)	***	1.7 (1.5, 2.0)	0.00 (0.00, 0.33)	0.06 (0.00, 4.46)
	mchc_blood_g_dl_min_value	67403 (61%)	60 (90%)	***	0.6 (0.5, 0.7)	33.00 (29.40, 35.40)	30.65 (27.44, 33.51)
	baso_blood_g_l_min_value	59405 (54%)	59 (88%)	***	1.6 (1.4, 1.9)	0.02 (0.00, 0.09)	0.05 (0.00, 0.54)
	baso_blood_g_l_variance	59405 (54%)	59 (88%)	***	1.6 (1.4, 1.8)	0.00 (0.00, 0.00)	0.00 (0.00, 0.08)
	mchc_blood_g_dl_max_value	67403 (61%)	60 (90%)	***	0.7 (0.6, 0.8)	34.00 (31.20, 36.60)	32.55 (29.55, 35.61)
	hgb_blood_g_dl_q25	109618 (100%)	67 (100%)	***	0.7 (0.6, 0.8)	13.10 (8.03, 16.50)	9.90 (6.53, 15.03)
	hgb_blood_g_dl_median	109618 (100%)	67 (100%)	***	0.7 (0.7, 0.8)	13.30 (8.60, 16.60)	10.20 (7.38, 15.12)
	hgb_blood_g_dl_q75	109618 (100%)	67 (100%)	***	0.7 (0.7, 0.8)	13.43 (9.10, 16.68)	10.45 (8.27, 15.63)
	rdw_cv_blood_percentage_variance	64716 (59%)	60 (90%)	***	1.4 (1.2, 1.5)	0.04 (0.00, 5.58)	0.85 (0.00, 11.87)
	mono_blood_percentage_variance	55032 (50%)	59 (88%)	***	1.4 (1.2, 1.6)	0.12 (0.00, 32.03)	2.33 (0.00, 58.38)
	rbc_blood_t_l_q25	109267 (99%)	65 (97%)	***	0.8 (0.7, 0.9)	4.36 (2.69, 5.49)	3.53 (2.09, 6.43)
	mpv_blood_fl_q75	98088 (89%)	56 (84%)	***	1.3 (1.2, 1.4)	10.38 (7.50, 12.80)	11.19 (9.05, 13.50)
	plt_blood_g_l_q75	109177 (99%)	65 (97%)	***	1.3 (1.1, 1.4)	241.00 (87.00, 502.00)	440.50 (37.20, 1699.00)
	mch_blood_pg_diff_first_last	46640 (42%)	33 (49%)	***	1.6 (1.3, 1.9)	0.40 (0.00, 3.90)	1.90 (0.08, 8.24)
	plt_blood_g_l_max_value	67240 (61%)	59 (88%)	***	1.3 (1.1, 1.4)	260.00 (92.00, 627.00)	475.00 (43.80, 2070.90)
	mch_blood_pg_variance	67450 (61%)	60 (90%)	***	1.3 (1.2, 1.4)	0.06 (0.00, 3.72)	0.42 (0.00, 14.59)
	hct_blood_percentage_q25	104144 (95%)	67 (100%)	***	0.8 (0.7, 0.9)	39.05 (24.45, 48.50)	31.23 (20.92, 47.57)
	hgb_blood_g_dl_min_value	67411 (61%)	60 (90%)	***	0.8 (0.7, 0.9)	12.20 (6.30, 16.20)	9.30 (5.50, 15.06)
	rbc_blood_t_l_min_value	67274 (61%)	59 (88%)	***	0.8 (0.7, 0.9)	4.08 (2.11, 5.40)	3.06 (1.70, 6.37)
	rbc_blood_t_l_median	109267 (99%)	65 (97%)	***	0.8 (0.7, 0.9)	4.41 (2.88, 5.51)	3.67 (2.29, 6.55)
	mcv_blood_fl_variance	67408 (61%)	60 (90%)	***	1.3 (1.1, 1.4)	0.61 (0.00, 26.09)	2.88 (0.00, 91.30)

mono_blood_percentage_min_value	55032 (50%)	59 (88%)	***	0.8 (0.7, 0.9)	7.00 (1.00, 14.10)	4.00 (0.95, 12.29)
mpv_blood_fl_median	98088 (89%)	56 (84%)	***	1.2 (1.1, 1.4)	10.25 (7.40, 12.65)	11.05 (8.95, 13.04)
mpv_blood_fl_max_value	60833 (55%)	50 (75%)	***	1.3 (1.1, 1.4)	10.60 (7.40, 13.40)	11.65 (9.17, 13.70)
hgb_blood_g_dl_max_value	67411 (61%)	60 (90%)	***	0.8 (0.7, 0.9)	13.70 (9.40, 17.00)	11.30 (8.54, 16.31)
plt_blood_g_l_median	109177 (99%)	65 (97%)	***	1.2 (1.1, 1.3)	233.00 (73.50, 468.00)	416.00 (33.00, 1418.00)
plt_blood_g_l_q25	109177 (99%)	65 (97%)	***	1.2 (1.1, 1.3)	226.00 (59.51, 445.00)	391.50 (22.50, 1015.10)
plt_blood_g_l_variance	67240 (61%)	59 (88%)	***	1.2 (1.1, 1.4)	259.00 (0.00, 15534.44)	1681.00 (0.00, 344681.78)
rdw_cv_blood_percentage_diff_first_last	42704 (39%)	33 (49%)	***	1.4 (1.2, 1.6)	0.30 (0.00, 4.90)	2.10 (0.08, 9.30)
baso_blood_percentage_diff_first_last	21179 (19%)	35 (52%)	***	1.5 (1.2, 1.9)	0.10 (0.00, 0.90)	0.30 (0.00, 2.77)
mono_blood_g_l_variance	59502 (54%)	58 (87%)	***	1.3 (1.1, 1.4)	0.00 (0.00, 0.30)	0.01 (0.00, 2.31)
hct_blood_percentage_median	104144 (95%)	67 (100%)	***	0.8 (0.8, 0.9)	39.50 (26.20, 48.70)	33.60 (23.05, 47.88)
rbc_blood_t_l_q75	109267 (99%)	65 (97%)	***	0.8 (0.8, 0.9)	4.47 (3.05, 5.55)	3.88 (2.52, 6.73)
baso_blood_g_l_diff_first_last	21164 (19%)	22 (33%)	***	2.0 (1.4, 3.0)	0.01 (0.00, 0.08)	0.03 (0.00, 1.07)
p_lcr_blood_percentage_q75	67444 (61%)	54 (81%)	***	1.2 (1.1, 1.3)	29.50 (16.10, 47.70)	34.31 (20.93, 48.81)
lymph_blood_percentage_q25	88541 (80%)	66 (99%)	**	0.8 (0.8, 0.9)	22.60 (4.30, 47.90)	15.43 (2.79, 30.73)
rbc_blood_t_l_max_value	67274 (61%)	59 (88%)	**	0.8 (0.8, 0.9)	4.55 (3.17, 5.69)	3.97 (2.68, 7.01)
hct_blood_percentage_min_value	64609 (59%)	60 (90%)	**	0.8 (0.8, 0.9)	36.60 (19.50, 47.90)	29.45 (17.75, 47.05)
mch_blood_pg_q25	109666 (100%)	67 (100%)	**	0.9 (0.8, 0.9)	30.05 (24.05, 34.32)	28.70 (21.44, 37.07)
plt_blood_g_l_min_value	67240 (61%)	59 (88%)	**	1.2 (1.1, 1.3)	205.00 (25.00, 423.00)	306.00 (12.45, 921.55)
hct_blood_percentage_q75	104144 (95%)	67 (100%)	**	0.9 (0.8, 0.9)	40.00 (27.75, 49.00)	35.00 (24.84, 49.91)
mcv_blood_fl_max_value	67408 (61%)	60 (90%)	**	1.2 (1.1, 1.3)	91.00 (78.20, 107.00)	94.75 (75.17, 118.52)
neut_blood_g_l_variance	57732 (52%)	57 (85%)	**	1.2 (1.1, 1.4)	0.01 (0.00, 28.95)	0.49 (0.00, 228.34)
pdw_blood_fl_q75	67219 (61%)	53 (79%)	**	1.2 (1.1, 1.3)	12.58 (9.20, 19.00)	14.02 (9.94, 26.17)
p_lcr_blood_percentage_median	67444 (61%)	54 (81%)	**	1.2 (1.1, 1.3)	28.80 (15.70, 46.60)	33.60 (19.45, 48.71)
mpv_blood_fl_q25	98088 (89%)	56 (84%)	**	1.2 (1.1, 1.3)	10.12 (7.30, 12.50)	10.50 (8.77, 12.96)
mono_blood_g_l_q75	96557 (88%)	64 (96%)	**	1.2 (1.1, 1.3)	0.63 (0.24, 1.68)	0.79 (0.24, 3.79)
lymph_blood_percentage_diff_first_last	21616 (20%)	48 (72%)	**	0.8 (0.7, 0.9)	3.00 (0.00, 26.80)	2.95 (0.22, 20.03)
lymph_blood_g_l_variance	59505 (54%)	58 (87%)	**	1.2 (1.1, 1.3)	0.00 (0.00, 1.00)	0.03 (0.00, 1.36)
lymph_blood_percentage_variance	54705 (50%)	59 (88%)	**	1.2 (1.1, 1.3)	0.51 (0.00, 192.00)	12.43 (0.00, 143.45)
mchc_blood_g_dl_variance	67403 (61%)	60 (90%)	**	1.2 (1.1, 1.3)	0.12 (0.00, 1.95)	0.35 (0.00, 2.12)
mcv_blood_fl_q75	109615 (100%)	67 (100%)	**	1.1 (1.0, 1.2)	89.90 (77.78, 103.00)	92.50 (75.04, 116.58)
mcv_blood_fl_diff_first_last	46609 (42%)	33 (49%)	*	1.2 (1.1, 1.4)	1.20 (0.00, 10.40)	2.60 (0.10, 21.30)
mpv_blood_fl_min_value	60833 (55%)	50 (75%)	*	1.2 (1.0, 1.3)	9.80 (7.00, 12.30)	10.10 (8.35, 13.03)
mch_blood_pg_min_value	67450 (61%)	60 (90%)	*	0.9 (0.8, 1.0)	29.70 (22.60, 34.10)	28.15 (19.77, 36.66)
mch_blood_pg_median	109666 (100%)	67 (100%)	*	0.9 (0.8, 1.0)	30.20 (24.40, 34.50)	29.02 (22.08, 38.11)
p_lcr_blood_percentage_max_value	38017 (35%)	48 (72%)	*	1.2 (1.0, 1.3)	31.80 (17.30, 51.60)	37.55 (20.99, 51.03)
p_lcr_blood_percentage_q25	67444 (61%)	54 (81%)	*	1.1 (1.0, 1.3)	28.10 (15.25, 45.80)	30.35 (17.78, 48.61)
rbc_blood_t_l_variance	67274 (61%)	59 (88%)	*	1.1 (1.0, 1.3)	0.03 (0.00, 0.50)	0.08 (0.00, 0.74)
lymph_blood_percentage_median	88541 (80%)	66 (99%)	*	0.9 (0.8, 1.0)	23.60 (4.90, 49.15)	16.75 (3.26, 41.29)
mono_blood_percentage_q25	89056 (81%)	66 (99%)	*	0.9 (0.8, 1.0)	7.80 (2.50, 14.70)	6.26 (2.00, 17.09)

eo_blood_percentage_diff_first_last	21528 (20%)	43 (64%)	*	0.8 (0.7, 1.0)	0.40 (0.00, 5.30)	0.50 (0.00, 3.33)
p_lcr_blood_percentage_min_value	38017 (35%)	48 (72%)	*	1.1 (1.0, 1.3)	26.70 (13.60, 45.00)	27.90 (15.49, 49.24)
hct_blood_percentage_variance	64609 (59%)	60 (90%)	*	1.1 (1.0, 1.3)	1.96 (0.00, 39.06)	4.55 (0.00, 47.06)
hct_blood_percentage_max_value	64609 (59%)	60 (90%)	*	0.9 (0.8, 1.0)	40.70 (28.80, 50.40)	36.20 (25.60, 52.71)
eo_blood_percentage_variance	54712 (50%)	59 (88%)	*	1.2 (1.0, 1.3)	0.01 (0.00, 7.70)	0.25 (0.00, 1.61)
pdw_blood_fl_max_value	36545 (33%)	47 (70%)	*	1.1 (1.0, 1.3)	13.30 (9.50, 21.50)	15.30 (10.16, 26.49)
mchc_blood_g_dl_diff_first_last	46637 (42%)	33 (49%)	*	1.2 (1.0, 1.4)	0.50 (0.00, 2.90)	1.10 (0.08, 2.34)
pdw_blood_fl_median	67219 (61%)	53 (79%)	*	1.1 (1.0, 1.2)	12.30 (9.10, 18.40)	13.30 (9.68, 25.40)
lymph_blood_percentage_min_value	54705 (50%)	59 (88%)	*	0.9 (0.8, 1.0)	18.90 (2.40, 47.20)	12.90 (2.09, 27.68)
plt_blood_g_l_diff_first_last	45654 (42%)	32 (48%)	*	1.2 (1.0, 1.4)	26.00 (0.00, 237.00)	59.00 (1.77, 1750.70)
mono_blood_g_l_max_value	59502 (54%)	58 (87%)	*	1.1 (1.0, 1.2)	0.70 (0.24, 2.35)	0.91 (0.26, 4.34)
eo_blood_percentage_max_value	54712 (50%)	59 (88%)	NS	1.1 (1.0, 1.2)	2.10 (0.00, 10.30)	3.00 (0.23, 7.55)
eo_blood_g_l_min_value	59208 (54%)	58 (87%)	NS	1.1 (1.0, 1.2)	0.07 (0.00, 0.48)	0.07 (0.00, 0.81)
lymph_blood_percentage_q75	88541 (80%)	66 (99%)	NS	0.9 (0.8, 1.0)	24.80 (5.35, 51.30)	19.05 (3.60, 48.08)
hgb_blood_g_dl_variance	67411 (61%)	60 (90%)	NS	1.1 (1.0, 1.2)	0.23 (0.00, 4.61)	0.50 (0.00, 4.10)
mono_blood_g_l_median	96557 (88%)	64 (96%)	NS	1.1 (1.0, 1.2)	0.60 (0.22, 1.55)	0.72 (0.22, 2.88)
eo_blood_g_l_q75	96078 (87%)	64 (96%)	NS	1.1 (1.0, 1.2)	0.12 (0.00, 0.58)	0.15 (0.00, 3.00)
eo_blood_g_l_q25	96078 (87%)	64 (96%)	NS	1.1 (1.0, 1.2)	0.10 (0.00, 0.51)	0.11 (0.00, 2.56)
eo_blood_g_l_median	96078 (87%)	64 (96%)	NS	1.1 (1.0, 1.2)	0.11 (0.00, 0.54)	0.12 (0.00, 2.75)
eo_blood_g_l_max_value	59208 (54%)	58 (87%)	NS	1.1 (1.0, 1.2)	0.14 (0.00, 0.77)	0.21 (0.00, 2.43)
mcv_blood_fl_median	109615 (100%)	67 (100%)	NS	1.1 (1.0, 1.2)	89.50 (77.10, 102.00)	91.55 (74.00, 116.14)
lymph_blood_g_l_q25	96561 (88%)	64 (96%)	NS	0.9 (0.8, 1.0)	1.61 (0.43, 3.72)	1.30 (0.54, 4.69)
pdw_blood_fl_min_value	36545 (33%)	47 (70%)	NS	1.1 (1.0, 1.2)	11.60 (8.50, 17.40)	12.10 (8.53, 24.67)
eo_blood_percentage_min_value	54712 (50%)	59 (88%)	NS	1.1 (1.0, 1.2)	0.90 (0.00, 6.10)	1.00 (0.00, 4.52)
eo_blood_g_l_variance	59208 (54%)	58 (87%)	NS	1.1 (1.0, 1.3)	0.00 (0.00, 0.04)	0.00 (0.00, 0.25)
pdw_blood_fl_q25	67219 (61%)	53 (79%)	NS	1.1 (1.0, 1.2)	12.10 (8.95, 18.00)	12.75 (9.21, 24.84)
neut_blood_percentage_diff_first_last	20472 (19%)	22 (33%)	NS	0.8 (0.7, 1.0)	3.90 (0.00, 32.60)	5.25 (0.10, 19.03)
mpv_blood_fl_variance	60833 (55%)	50 (75%)	NS	1.1 (1.0, 1.2)	0.05 (0.00, 1.28)	0.14 (0.00, 1.53)
eo_blood_percentage_q75	88546 (80%)	66 (99%)	NS	1.1 (1.0, 1.2)	1.68 (0.00, 7.50)	2.00 (0.06, 6.05)
hgb_blood_g_dl_diff_first_last	46627 (42%)	33 (49%)	NS	1.1 (1.0, 1.3)	0.74 (0.00, 4.70)	1.20 (0.16, 5.02)
neut_blood_g_l_q75	94533 (86%)	63 (94%)	NS	1.1 (1.0, 1.2)	4.90 (1.71, 16.73)	6.34 (1.72, 72.94)
mpv_blood_fl_diff_first_last	39724 (36%)	20 (30%)	NS	1.1 (0.9, 1.4)	0.30 (0.00, 2.20)	0.55 (0.20, 2.45)
mcv_blood_fl_q25	109615 (100%)	67 (100%)	NS	1.1 (1.0, 1.2)	89.10 (76.20, 101.30)	90.70 (73.47, 111.77)
neut_blood_percentage_variance	53295 (48%)	58 (87%)	NS	1.1 (1.0, 1.2)	0.90 (0.00, 292.07)	12.41 (0.00, 140.18)
mcv_blood_fl_min_value	67408 (61%)	60 (90%)	NS	1.1 (1.0, 1.2)	88.30 (73.40, 100.70)	88.55 (68.24, 112.58)
lymph_blood_g_l_median	96561 (88%)	64 (96%)	NS	0.9 (0.9, 1.0)	1.67 (0.50, 3.82)	1.46 (0.70, 5.03)
p_lcr_blood_percentage_variance	38017 (35%)	48 (72%)	NS	0.9 (0.8, 1.0)	1.96 (0.00, 44.17)	2.76 (0.00, 50.19)
hct_blood_percentage_diff_first_last	43920 (40%)	33 (49%)	NS	1.1 (0.9, 1.3)	2.20 (0.00, 13.60)	3.60 (0.50, 16.02)
eo_blood_percentage_median	88546 (80%)	66 (99%)	NS	1.1 (1.0, 1.1)	1.50 (0.00, 6.90)	1.80 (0.06, 5.28)
mch_blood_pg_q75	109666 (100%)	67 (100%)	NS	1.0 (0.9, 1.0)	30.30 (24.76, 34.82)	29.95 (22.34, 39.36)

	lymph_blood_g_l_min_value	59505 (54%)	58 (87%)	NS	0.9 (0.9, 1.0)	1.41 (0.25, 3.61)	1.12 (0.36, 3.53)
	wbc_blood_g_l_q75	109181 (99%)	65 (97%)	NS	1.1 (1.0, 1.1)	8.09 (3.80, 22.21)	8.63 (3.61, 100.33)
	mono_blood_g_l_q25	96557 (88%)	64 (96%)	NS	1.0 (1.0, 1.1)	0.59 (0.18, 1.48)	0.65 (0.19, 2.88)
	mono_blood_percentage_max_value	55032 (50%)	59 (88%)	NS	1.0 (1.0, 1.1)	9.00 (3.40, 24.60)	9.90 (2.91, 27.27)
	neut_blood_g_l_min_value	57732 (52%)	57 (85%)	NS	1.0 (0.9, 1.0)	4.09 (0.49, 14.78)	3.60 (0.39, 36.20)
	neut_blood_percentage_min_value	53295 (48%)	58 (87%)	NS	1.0 (0.9, 1.0)	60.20 (11.80, 88.00)	61.50 (20.56, 83.49)
	neut_blood_g_l_median	94533 (86%)	63 (94%)	NS	1.0 (1.0, 1.1)	4.68 (1.58, 15.48)	5.53 (1.14, 65.16)
	rbc_blood_t_l_diff_first_last	45997 (42%)	32 (48%)	NS	1.1 (0.9, 1.2)	0.25 (0.00, 1.53)	0.45 (0.00, 2.15)
	mono_blood_percentage_median	89056 (81%)	66 (99%)	NS	1.0 (0.9, 1.1)	8.10 (3.00, 15.40)	7.60 (2.40, 19.14)
	neut_blood_percentage_max_value	53295 (48%)	58 (87%)	NS	1.0 (0.9, 1.1)	69.50 (37.30, 93.50)	71.55 (45.88, 89.76)
	mono_blood_g_l_diff_first_last	20776 (19%)	22 (33%)	NS	1.1 (0.9, 1.3)	0.08 (0.00, 1.02)	0.18 (0.02, 4.90)
	eo_blood_percentage_q25	88546 (80%)	66 (99%)	NS	1.0 (0.9, 1.1)	1.30 (0.00, 6.60)	1.32 (0.00, 4.73)
	wbc_blood_g_l_median	109181 (99%)	65 (97%)	NS	1.0 (0.9, 1.1)	7.67 (3.55, 19.33)	8.00 (2.75, 72.95)
	neut_blood_g_l_max_value	57732 (52%)	57 (85%)	NS	1.0 (0.9, 1.1)	5.64 (1.75, 22.73)	6.59 (1.93, 77.53)
	pdw_blood_fl_diff_first_last	30740 (28%)	19 (28%)	NS	1.1 (0.9, 1.3)	0.60 (0.00, 4.90)	1.00 (0.05, 4.50)
	lymph_blood_g_l_max_value	59505 (54%)	58 (87%)	NS	1.0 (0.9, 1.1)	1.81 (0.57, 4.92)	1.79 (0.75, 5.41)
	wbc_blood_g_l_min_value	67226 (61%)	59 (88%)	NS	1.0 (0.9, 1.1)	6.54 (1.75, 16.10)	6.30 (1.17, 42.89)
	mono_blood_percentage_q75	89056 (81%)	66 (99%)	NS	1.0 (0.9, 1.1)	8.40 (3.27, 16.77)	8.40 (2.42, 21.74)
	mono_blood_g_l_min_value	59502 (54%)	58 (87%)	NS	1.0 (0.9, 1.1)	0.53 (0.07, 1.45)	0.52 (0.10, 1.78)
	neut_blood_percentage_q25	87062 (79%)	65 (97%)	NS	1.0 (0.9, 1.1)	62.90 (31.80, 88.30)	64.60 (30.27, 86.08)
	neut_blood_percentage_q75	87062 (79%)	65 (97%)	NS	1.0 (0.9, 1.1)	65.60 (36.90, 89.90)	67.45 (45.05, 87.80)
	lymph_blood_g_l_diff_first_last	20776 (19%)	22 (33%)	NS	1.0 (0.8, 1.2)	0.20 (0.00, 2.00)	0.32 (0.04, 3.02)
	wbc_blood_g_l_diff_first_last	45795 (42%)	31 (46%)	NS	1.0 (0.9, 1.2)	1.43 (0.00, 14.47)	2.07 (0.05, 43.90)
	mono_blood_percentage_diff_first_last	21836 (20%)	47 (70%)	NS	1.0 (0.9, 1.2)	1.00 (0.00, 9.80)	1.80 (0.20, 6.67)
	p_lcr_blood_percentage_diff_first_last	27406 (25%)	16 (24%)	NS	1.0 (0.8, 1.3)	1.90 (0.00, 13.00)	2.50 (0.50, 15.20)
	mch_blood_pg_max_value	67450 (61%)	60 (90%)	NS	1.0 (0.9, 1.1)	30.60 (24.90, 36.10)	30.40 (22.24, 40.48)
	neut_blood_percentage_median	87062 (79%)	65 (97%)	NS	1.0 (0.9, 1.1)	64.30 (35.01, 89.00)	64.90 (41.18, 86.91)
	wbc_blood_g_l_q25	109181 (99%)	65 (97%)	NS	1.0 (0.9, 1.1)	7.30 (3.16, 17.50)	7.36 (1.66, 66.87)
	wbc_blood_g_l_max_value	67226 (61%)	59 (88%)	NS	1.0 (0.9, 1.1)	9.55 (4.01, 35.82)	9.16 (3.83, 98.48)
	eo_blood_g_l_diff_first_last	20643 (19%)	22 (33%)	NS	1.0 (0.8, 1.2)	0.02 (0.00, 0.40)	0.05 (0.01, 1.61)
	lymph_blood_percentage_max_value	54705 (50%)	59 (88%)	NS	1.0 (0.9, 1.1)	26.60 (5.50, 68.60)	25.70 (5.18, 64.56)
	neut_blood_g_l_diff_first_last	20451 (19%)	22 (33%)	NS	1.0 (0.8, 1.2)	0.71 (0.00, 10.05)	1.00 (0.11, 46.54)
	neut_blood_g_l_q25	94533 (86%)	63 (94%)	NS	1.0 (0.9, 1.1)	4.48 (1.39, 14.71)	4.57 (0.51, 60.95)
	wbc_blood_g_l_variance	67226 (61%)	59 (88%)	NS	1.0 (0.9, 1.1)	0.90 (0.00, 63.86)	0.87 (0.00, 365.70)
	pdw_blood_fl_variance	36545 (33%)	47 (70%)	NS	1.0 (0.9, 1.1)	0.25 (0.00, 6.69)	0.37 (0.00, 14.12)
	lymph_blood_g_l_q75	96561 (88%)	64 (96%)	NS	1.0 (0.9, 1.1)	1.73 (0.55, 3.98)	1.60 (0.73, 5.50)
Discrete laboratory	eo_blood_percentage_mono_first_last	10922 (10%)	26 (39%)	***	4.0 (2.4, 6.6)	-	-
	baso_blood_percentage_mono_first_last	8373 (8%)	19 (28%)	***	3.3 (1.9, 5.6)	-	-
	lymph_blood_percentage_mono_first_last	10592 (10%)	22 (33%)	***	3.1 (1.8, 5.3)	-	-
	mono_blood_percentage_mono_first_last	11362 (10%)	18 (27%)	*	2.1 (1.2, 3.7)	-	-

	plt_blood_g_l_mono_first_last	19869 (18%)	8 (12%)	*	0.4 (0.2, 0.8)	-	-
	baso_blood_g_l_mono_first_last	6935 (6%)	11 (16%)	NS	2.0 (1.0, 3.8)	-	-
	mpv_blood_fl_mono_first_last	19371 (18%)	10 (15%)	NS	0.5 (0.3, 1.0)	-	-
	hct_blood_percentage_mono_first_last	16228 (15%)	21 (31%)	NS	1.7 (1.0, 2.9)	-	-
	hgb_blood_g_dl_mono_first_last	16705 (15%)	21 (31%)	NS	1.6 (1.0, 2.8)	-	-
	mchc_blood_g_dl_mono_first_last	21360 (19%)	25 (37%)	NS	1.5 (0.9, 2.6)	-	-
	eo_blood_g_l_mono_first_last	9927 (9%)	13 (19%)	NS	1.6 (0.9, 3.0)	-	-
	wbc_blood_g_l_mono_first_last	21633 (20%)	14 (21%)	NS	0.6 (0.4, 1.2)	-	-
	lymph_blood_g_l_mono_first_last	9559 (9%)	12 (18%)	NS	1.5 (0.8, 2.9)	-	-
	rdw_cv_blood_percentage_mono_first_last	22842 (21%)	16 (24%)	NS	0.7 (0.4, 1.3)	-	-
	rbc_blood_t_l_mono_first_last	17097 (16%)	19 (28%)	NS	1.4 (0.8, 2.4)	-	-
	mono_blood_g_l_mono_first_last	10311 (9%)	12 (18%)	NS	1.4 (0.7, 2.6)	-	-
	neut_blood_g_l_mono_first_last	9456 (9%)	11 (16%)	NS	1.4 (0.7, 2.7)	-	-
	mch_blood_pg_mono_first_last	20987 (19%)	22 (33%)	NS	1.3 (0.8, 2.2)	-	-
	p_lcr_blood_percentage_mono_first_last	14490 (13%)	10 (15%)	NS	0.7 (0.4, 1.4)	-	-
	mcv_blood_fl_mono_first_last	23190 (21%)	18 (27%)	NS	0.8 (0.5, 1.4)	-	-
	pdw_blood_fl_mono_first_last	15575 (14%)	13 (19%)	NS	0.9 (0.5, 1.7)	-	-
	neut_blood_percentage_mono_first_last	9886 (9%)	9 (13%)	NS	1.0 (0.5, 2.1)	-	-
Phenotypes	Other anemias	1554 (1%)	10 (15%)	***	8.5 (4.3, 16.8)	-	-
	Anemia	2215 (2%)	9 (13%)	***	5.2 (2.6, 10.6)	-	-
	Other ill-defined and unknown causes of morbidity a	1565 (1%)	5 (7%)	**	3.9 (1.5, 9.6)	-	-
	Chemotherapy	4145 (4%)	7 (10%)	NS	2.0 (0.9, 4.5)	-	-
	Hypertension	14401 (13%)	12 (18%)	NS	0.9 (0.5, 1.7)	-	-
ICD-10 codes	Z03	3118 (3%)	19 (28%)	***	9.5 (5.5, 16.4)	-	-
	D64.8	1058.0 (<1%)	8 (12%)	***	9.7 (4.6, 20.4)	-	-
	D64.9	2215 (2%)	9 (13%)	***	5.2 (2.6, 10.6)	-	-
	R69	1565 (1%)	5 (7%)	**	3.9 (1.5, 9.6)	-	-
	Z51.1	4138 (4%)	7 (10%)	NS	2.0 (0.9, 4.5)	-	-
	Z03.9	5424 (5%)	6 (9%)	NS	1.3 (0.6, 3.0)	-	-
	I10	14401 (13%)	12 (18%)	NS	0.9 (0.5, 1.7)	-	-
